# Multi-Omic analyses characterize the ceramide/sphingomyelin pathway as a therapeutic target in Alzheimer’s disease

**DOI:** 10.1038/s42003-022-04011-6

**Published:** 2022-10-08

**Authors:** Priyanka Baloni, Matthias Arnold, Luna Buitrago, Kwangsik Nho, Herman Moreno, Kevin Huynh, Barbara Brauner, Gregory Louie, Alexandra Kueider-Paisley, Karsten Suhre, Andrew J. Saykin, Kim Ekroos, Peter J. Meikle, Leroy Hood, Nathan D. Price, The Alzheimer’s Disease Metabolomics Consortium, Matthias Arnold, Colette Blach, Rima Kaddurah-Daouk, Murali Doraiswamy, Siamak Mahmoudiandehkordi, Kathleen Welsh-Bohmer, Brenda Plassman, Jan Krumsiek, Richa Batra, Andrew Saykin, Jingwen Yan, Shannon L. Risacher, Peter Meikle, Tingting Wang, Arfan Ikram, Shahzad Ahmad, Thomas Hankemeier, Ivan A. Hernandez, Almut Heinken, Filippo Martinelli, Ines Thiele, Johannes Hertel, Tim Hensen, Tim Hulshof, Lindsay A. Farrer, Rhoda Au, Wendy Wei Qiao Qiu, Thor Stein, Naama Karu, Kamil Borkowski, John Newman, Wei Jia, Guoxiang Xie, Jingye Wang, Runmin Wei, Dan Rader, Mitchel Kling, Leslie Shaw, P. Murali Doraiswamy, Cory C. Funk, A. Iván Hernández, Gabi Kastenmüller, Rebecca Baillie, Xianlin Han, Rima Kaddurah-Daouk

**Affiliations:** 1 Institute for Systems Biology, Seattle, WA USA; 2 School of Health Sciences, Purdue University, West Lafayette, IN USA; 3 Institute of Computational Biology, Helmholtz Zentrum München - German Research Center for Environmental Health, Neuherberg, Germany; 4 Department of Psychiatry and Behavioral Sciences, Duke University School of Medicine, Durham, Durham, NC USA; 5 Department of Neurology/Pharmacology, SUNY Downstate Medical Center, Brooklyn, NY USA; 6 Indiana Alzheimer’s Disease Research Center and Department of Radiology and Imaging Sciences, Indiana University School of Medicine, Indianapolis, IN USA; 7 Metabolomics Laboratory, Baker Heart and Diabetes Institute, Melbourne, VIC Australia; 8 Department of Physiology and Biophysics, Weill Cornell Medicine-Qatar, Education City, PO 24144 Doha, Qatar; 9 Lipidomics Consulting Ltd., Esbo, Finland; 10 Department of Pathology, SUNY Downstate Medical Center, Brooklyn, NY USA; 11 Rosa & Co LLC, San Carlos, CA USA; 12 University of Texas Health Science Center at San Antonio, San Antonio, TX USA; 13 Department of Medicine, Duke University, Durham, NC USA; 14 Duke Institute of Brain Sciences, Duke University, Durham, NC USA; 15 Institute for Computational Biomedicine, Weill Cornell Medicine, New York, NY USA; 16 Department of Epidemiology, ErasmusMC, Rotterdam, The Netherlands; 17 Division of Analytical Biosciences, Leiden/Amsterdam Center for Drug Research, Leiden University, Leiden, The Netherlands; 18 National University of, Ireland Galway, Ireland; 19 Boston University, Boston, MA USA; 20 University of Alberta, Edmonton, AB Canada; 21 University of California, Davis, CA USA; 22 University of Hawaii-Manoa, Honolulu, HI USA; 23 University of Pennsylvania, Philadelphia, PA USA

**Keywords:** Neuroscience, Alzheimer's disease

## Abstract

Dysregulation of sphingomyelin and ceramide metabolism have been implicated in Alzheimer’s disease. Genome-wide and transcriptome-wide association studies have identified various genes and genetic variants in lipid metabolism that are associated with Alzheimer’s disease. However, the molecular mechanisms of sphingomyelin and ceramide disruption remain to be determined. We focus on the sphingolipid pathway and carry out multi-omics analyses to identify central and peripheral metabolic changes in Alzheimer’s patients, correlating them to imaging features. Our multi-omics approach is based on (a) 2114 human post-mortem brain transcriptomics to identify differentially expressed genes; (b) in silico metabolic flux analysis on context-specific metabolic networks identified differential reaction fluxes; (c) multimodal neuroimaging analysis on 1576 participants to associate genetic variants in sphingomyelin pathway with Alzheimer’s disease pathogenesis; (d) plasma metabolomic and lipidomic analysis to identify associations of lipid species with dysregulation in Alzheimer’s; and (e) metabolite genome-wide association studies to define receptors within the pathway as a potential drug target. We validate our hypothesis in amyloidogenic APP/PS1 mice and show prolonged exposure to fingolimod alleviated synaptic plasticity and cognitive impairment in mice. Our integrative multi-omics approach identifies potential targets in the sphingomyelin pathway and suggests modulators of S1P metabolism as possible candidates for Alzheimer’s disease treatment.

## Abstract (web summary)

Complementary multi-omics approach, in silico metabolic flux, and neuroimaging analyses link the sphingolipid pathway to Alzheimer’s disease pathogenesis with therapeutic targets validated using behavioral assessments in amyloidogenic APP/PS1 mice.

## Introduction

To date, ~400 trials of experimental Alzheimer’s treatments have failed^1^. In the wake of such large-scale failure, additional hypotheses have been proposed to accelerate strategies for treatment and researchers are pursuing alternative approaches, with a greater focus on the complex mechanisms underlying this neurodegenerative disease^2^. In an effort to address this knowledge gap, the NIH-funded Accelerating Medicines Partnership—Alzheimer’s Disease (AMP-AD) has successfully generated new hypotheses and insights around Alzheimer’s disease (AD) and produced large, publicly available datasets. The knowledge gained from this initiative will lead to a major paradigm shift in research focus, resulting in novel targets and testable hypotheses that are currently being investigated in clinical phase 1 and 2 trials aimed at neuroprotection and anti-neuroinflammation^3^. These new hypotheses also suggest potential drug repositioning and development.

While the central neuropathological features of AD are the accumulation of misfolded β-amyloid (Aβ) plaques and phosphorylated tau proteins, brain atrophy and neuronal loss are equally important. The relationship between Aβ accumulation, tau phosphorylation, and neuronal loss is unclear. What is clear is that AD etiology is multifactorial, with genetic contributions, protein mis-trafficking and turnover, altered glucose metabolism, and lipid metabolism failures^4^. Recent studies have clarified the important relationship between the immune system and lipid metabolism and more than half of the genes implicated in AD via genetic association screens are linked to lipid metabolism and inflammation^5^. Exploring how these genes factor into AD pathophysiology over the last few years is starting to increase our understanding of the role of lipid metabolism in AD. APOE4, the strongest genetic risk factor for late-onset AD, is centrally involved in lipid metabolism, including the transport of cholesterol to neurons from astrocytes^6^. Additionally, several independent genetic association studies have reported replicable associations of the *APOE* locus with blood levels of sphingolipid species^7–9^. Lipids, including sphingomyelins (SMs), have been shown to be disrupted in AD^10–12^. However, few studies have taken a holistic view of how lipid dysregulation contributes to AD pathogenesis.

Brain lipids constitute ~50% of the brain’s dry weight with myelin, a proteolipid, composed of 70–80% lipids^13^. Several lines of supporting evidence implicate various sphingolipids in neuronal signaling and toxicity^14,15^. Sphingomyelin (SM) primarily resides in two locations within the brain: (1) lipid rafts, found in neurons, astrocytes, and microglia where they are involved in several aspects of signal transduction and homeostasis of the brain and (2) the membranous myelin sheath that insulates many nerve cell axons^16^. As part of lipid rafts, SMs are involved in signal transduction and the regulation of inflammatory processes and response to oxidative stress^17^. Our previous studies^18–21^ indicated a complex pattern of deregulation in the sphingolipid metabolism, including ceramides, in the early stages of AD. We have also reported changes at the gene expression level of the myelin network in AD^22^. Hydrolysis of sphingomyelin produces ceramide (Cer). Ceramides are the simplest of sphingolipids, are neurotoxic, and induce apoptosis^23,24^. Ceramides are known to mediate the relationship between Aβ and neurodegeneration^25^. Increasing Aβ levels elevate SM phosphodiesterase (SMase) activity leading to an increase in Cer^26,27^. It is suggested that the increase in ceramides boosts BACE-1 activity^28^, which cleaves amyloid precursor protein (APP) in two soluble Aβ. Sphingosine-1-phosphate (S1P), is an important neuroprotective signaling molecule and product of the SM pathway that blocks SMase activity^29^ and inhibits APP secretion^30^. By understanding the changes in SM/Cer homeostasis and their underlying mechanism, we can better understand how perturbations in the SM pathway contribute to neurodegeneration.

As part of normal homeostasis, microglia constantly surveil the brain parenchyma. In development, and throughout the normal lifespan, they remove neuronal synapses, eliminate dying neurons, and clean up myelin debris^31–34^. Sphingolipid-rich neuronal and myelin membranes captured through these processes undergo lysosomal degradation within microglia. This degradative process is facilitated by a lipid-sensing receptor, TREM2, that is activated by various lipids (including sphingolipids, sphingomyelin, and sulfatide). TREM2-deficient microglia phagocytose myelin debris but fail to clear myelin cholesterol, resulting in cholesteryl ester (CE) accumulation. CE increase is also observed in APOE-deficient glial cells, reflecting impaired brain cholesterol transport^35^. Recent studies have begun to elucidate the important role of microglia in AD, with evidence for differences in microglial subpopulations, related to myelin clearance and activation^36–42^.

This comprehensive study analyzed human in vivo data and post-mortem brain data to finely characterize the SM pathway for molecular links to AD pathogenesis. We used multi-omics approach to identify metabolic readouts that helped to characterize molecular changes back to potential intervention targets, which were experimentally validated in animal models resulting in repurposed drug for AD. By using complementary approaches, we were able to reveal how sphingosine 1-phosphate (S1P) regulates the balance in the pathway. We tested our hypothesis and demonstrated that fingolimod, an S1P receptor (S1PR) modulator, is able to improve cognition in amyloidogenic mice model. We highlight S1P as the metabolite involved in maintaining the balance in the pathway and identifying drugs regulating S1P levels that can be repurposed for AD.

## Results

### Post-mortem brain transcriptome analysis identifies global dysregulation of the SM pathway in AD

**Fig. 1:**
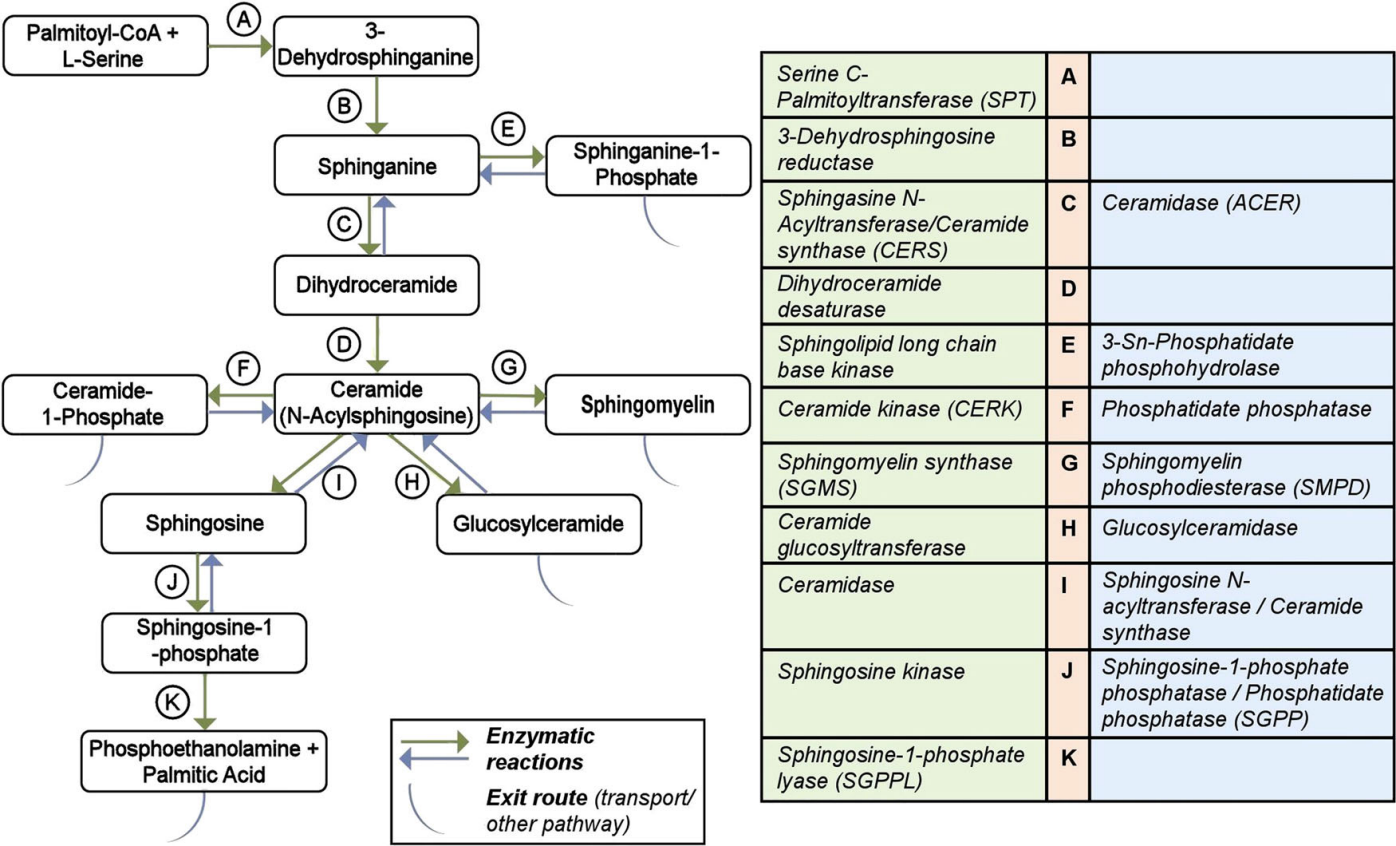
Overview of sphingolipid pathway manually curated from the Recon3D model. The metabolites participating in reactions are represented in boxes. The arrows for reactions A–K are colored based on the direction in the pathway. Some reactions are not reversible (single arrows). The table on the right lists the catalyzing enzymes in the sphingolipid pathway in humans and is denoted with the same color code as the reaction arrow.

We analyzed gene expression changes of well-characterized enzymes in the sphingolipid pathway from post-mortem brain RNA-seq data generated on seven brain regions (cerebellum, temporal cortex, dorsolateral prefrontal cortex, parahippocampal gyrus, frontal pole, inferior frontal gyrus, and superior temporal gyrus) in three independent cohorts (ROS/MAP, Mayo, and Mount Sinai), of 2114 brain samples as well as the cross-region, cross-study meta-analysis^43^. For this study, we manually curated the sphingolipid subsystem definition of the human genome-scale metabolic reconstruction^44^ resulting in the identification of a set of 35 enzymes catalyzing 18 enzymatic reactions within the SM pathway (Fig. 1, Supplementary Table 1). The reactions cover Cer and SM biosynthesis, as well as four exit routes (through sphinganine-1-phosphate, ceramide-1-phosphate, sphingomyelin, glycosphingolipids, and sphingosine-1-phosphate). Gene expression data were available for 31 of the 35 genes, the exceptions being *CERS3, ACER1, ASAH2*, and *ENPP7*. Low and/or no expression of these genes in the brain was confirmed in the GTEx Portal^45^.

Analysis of differential gene expression showed significant (FDR-corrected) gene expression changes in brain tissue of AD cases vs. controls for 20 of the genes (Supplementary Table 2). Of those 20, 19 showed differential expression in one or more studies/brain regions. Fourteen of these were also detected in the meta-analysis. Transcripts of *SPTLC3* were not measured in all brain regions, hence it was not reported in the meta-analysis. *DEGS1*, on the other hand, was insignificantly but consistently upregulated in the single studies, leading to a detectable significant overall upregulation in the meta-analysis. Almost all of the genes showed significantly higher expression in AD cases, consistent across all brain regions. Exceptions were *CERS5* (lower levels in cerebellum of AD cases; not significant in the meta-analysis), *CERS6* (higher levels in cerebellum vs. lower levels in the parahippocampal gyrus of AD cases; not significant in the meta-analysis), and *SMPD3* (lower levels in the temporal cortex of AD cases; also significant in the meta-analysis).

### In silico metabolic flux analysis identified reactions with differential fluxes in AD and control samples

**Fig. 2:**
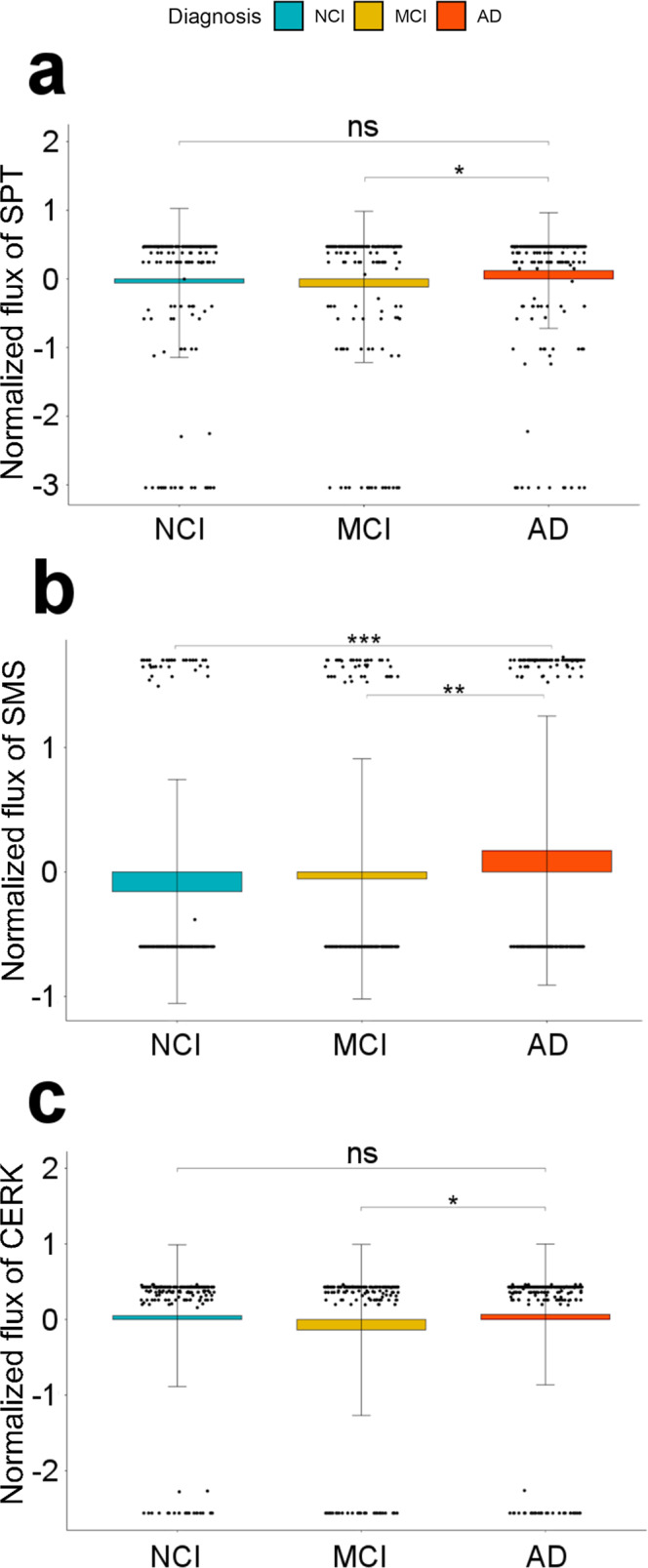
In silico flux analysis for metabolic reactions in the sphingolipid pathway. Box plot of normalized reaction fluxes for **a** serine palmitoyl transferase (SPT), **b** sphingomyelin synthase (SMS), and **c** ceramide kinase (CERK) reactions. The orange, mustard yellow, and blue bars correspond to Alzheimer’s Disease (AD), mild cognitive impairment (MCI), and no cognitive impairment (NCI). ∗*p* < 0.05, ∗∗*p* < 0.01, ∗∗∗*p* < 0.001, and ns is non-significant.

We used brain region-specific metabolic reconstructions^46^ and integrated the post-mortem brain RNA-seq data with them to identify reactions that had differential fluxes in AD vs. no cognitive impairment (NCI) or control individuals and mild cognitive impairment (MCI). For the dorsolateral prefrontal cortex, we identified reactions catalyzed by serine palmitoyltransferase (SPT, encoded by *SPTLC1/2/3*, enzyme A in Fig. 1), sphingomyelin synthase (SMS, encoded by *SGMS1/2*, enzyme G in Fig. 1), and ceramide kinase (CERK, encoded by *CERK*, enzyme F in Fig. 1) as having significant flux differences as shown in Fig. 2. The plots are colored based on the diagnosis and adjusted *p*-values are indicated. SPT catalyzes the first step in the biosynthesis of sphingolipids condensing serine and palmitoyl-CoA to form 3-ketosphinganine, which is the rate-limiting step in the synthesis of SMs (Fig. 1). For this reaction, we found significant differences in flux values comparing AD and mild cognitive impairment (MCI) cases (Fig. 2a; Supplementary Table 2). Sphingomyelin synthase synthesizes sphingomyelin from ceramide. Here, we observed AD samples having higher reaction fluxes compared to the NCI samples (Fig. 2b; Supplementary Table 2). We further identified flux differences for the reaction catalyzed by ceramide kinase (phosphorylation of ceramide to form ceramide-1-phosphate) in AD and NCI samples (Fig. 2c; Supplementary Table 2) and observed a significant difference between AD and MCI samples.

### Genetic association studies and multimodal neuroimaging analysis link SM pathway to AD pathogenesis

Using gene-based association analysis in 1576 participants of the AD neuroimaging initiative (ADNI) phases 1, GO and 2, we identified genetic variants in the coding regions linked to seven of the 35 genes in the SM pathway to be significantly associated with AD and its (bio)markers, which covered the whole spectrum of Amyloid, Tau, Neurodegeneration, Cognition (A-T-N-C) measures^47^ (Supplementary Table 3). A-T-N-C measures of AD are calculated by investigating genetic associations of CSF biomarker levels, brain atrophy (magnetic resonance imaging), brain glucose metabolism ([^18^F]FDG-PET), cognition, and clinical diagnosis. In this analysis, Bonferroni-significance was determined by gene-specific thresholds correcting for the number of all genetic variants assigned to a certain gene. Associated markers included CSF Aβ_1–42_ (*CERS2*, enzyme *C* in Fig. 1), the ratio between CSF tau (both total tau and p-tau) and CSF Aβ_1–42_ (*ACER2 (*enzyme C in Fig. 1), *PLPP2*), region of interest-based measures of [^18^F] fluorodeoxyglucose positron emission tomography (FDG-PET; *CERS3, SPHK2*), cognitive performance measured, among other, by the 13-item cognitive subscale of the AD assessment scale (ADAS-Cog.13; *CERS6, DEGS1*), and clinical AD (*CERS3, CERS6, DEGS1*). Furthermore, a detailed whole brain analysis of brain glucose metabolism (FDG-PET) on voxel-wise levels showed that rs1847325 in *CERS3* (enzyme C in Fig. 1) and rs281380 in *SPHK2* (J in Fig. 1) were significantly associated with increased brain glucose metabolism in the bilateral frontal, parietal, and temporal lobes (colored regions with corrected *p*-value < 0.05; Supplementary Fig. 1). Previously, a study on clinico-pathologic AD dementia^48^ yielded an association with *SMPD2* (enzyme G in Fig. 1) that is Bonferroni-significant at the gene-wide level.

**Fig. 3:**
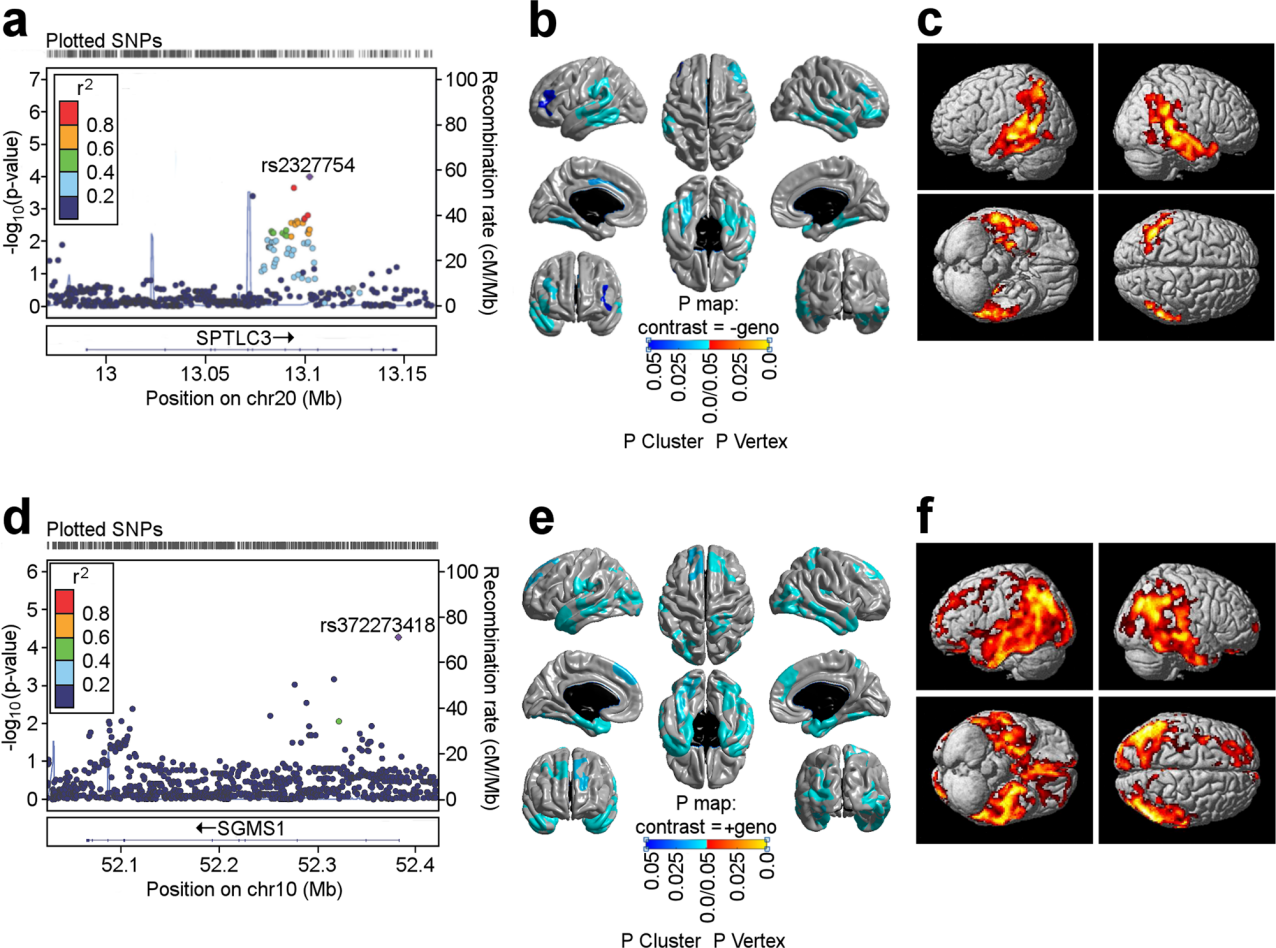
Association of genetic variants in *SPTLC3* and *SGMS1* with structural (MRI) and molecular (FDG-PET) neuroimaging phenotypes. **a** Gene-based association analysis of *SPTLC3* with cognitive performance (Rey auditory verbal learning test total score). **d** Gene-based association analysis of *SGMS1* with global brain glucose metabolism. **b** and **e** Surface-based whole brain analysis of cortical thickness (brain atrophy measured from MRI scans) for *SPTLC3* and *SGMS1*. **c** and **f** Voxel-based whole brain analysis of brain glucose metabolism measured from FDG-PET scans for *SPTLC3* and *SGMS1*. Red color suggests a decrease in glucose metabolism. chr chromosome, FDG fluorodeoxyglucose, MRI magnetic resonance imaging, PET positron emission tomography, SNP single nucleotide polymorphism.

A less stringent *p*-value cutoff (adjusting for multiple testing by permutation as SNPs are correlated due to linkage disequilibrium) identified variants in two additional genes, *SPTLC3* (enzyme A in Fig. 1) and *SGMS1* (enzyme G in Fig. 1). *SPTLC3* was associated with cognitive performance (corrected *p*-value = 0.02; Fig. 3a), brain atrophy in focal regions of the bilateral temporal and frontal lobes (determined by detailed surface-based whole-brain analysis of cortical thickness measured from MRI scans on a vertex-wise level; colored regions with corrected *p*-value < 0.05; Fig. 3b) and FDG-PET measures in the bilateral temporal and parietal lobes (colored regions with corrected *p*-value < 0.05; Fig. 3c). *SGMS1* was associated with brain glucose metabolism measured by region of interest-based FDG-PET (corrected *p*-value = 0.02; Fig. 3d) that was mapped by whole brain analysis to the bilateral temporal, parietal, and frontal lobes, as well as the hippocampus (colored regions with corrected *p*-value < 0.05; Fig. 3f). In addition, surface-based whole brain association analysis showed a significant association with cortical thickness in the bilateral temporal, parietal, and frontal lobes, with the strongest association located in the entorhinal cortex (colored regions with corrected *p*-value < 0.05; Fig. 3e).

### SM (d34:1)/SM (d43:1) ratio as a strong intermediate trait for sphingolipid dysregulation in AD

Sphingomyelin species (SMs) of differing lengths have been implicated in the early vs. late stages of AD20. SM (d34:1) is associated with CSF Aβ1-42 pathology, while SMs with longer fatty acid chains (≥C20) are correlated with brain atrophy and cognitive decline. Utilizing the concept of metabolite ratios^49^, which enables both removal of potentially remaining technical variance and modeling of enzymatic/pathway activity^9^, we selectively screened ratios of shorter chain SMs (<C20) and longer chain SMs (≥C20) in the ADNI-1 dataset (*n* = 732) similar to Toledo et al.^20^. This revealed the ratio of SM (d34:1) and SM (d43:1) as the metabolic trait most significantly associated with a diagnosis of clinical AD (*p*-value = 1.70 × 10^−4^, Pgain = 178.37), brain atrophy in regions implicated in AD^50^(*p*-value = 7.64 × 10^−6^, Pgain = 687.57) as well as cognition (measured by ADAS-Cog. 13; *p*-value = 4.36 × 10^−6^, Pgain = 2544.51). The modified Alzheimer’s Disease Assessment Scale cognitive subscale (ADAS-Cog 13-item scale)^51^ has all the original ADAS-Cog items with additional items that were aimed to increase the number of cognitive domains and range of symptom severity.

To expand upon and further validate this finding, we examined the same cohort (ADNI1) using a more comprehensive lipidomics method covering a broader range of sphingolipids. In total, 112 sphingolipids were examined in serum samples (*n* = 754), where chromatography enabled the separation of some isomeric and isobaric species. Regression analysis (adjusting for age, sex, BMI, HDL-C, total cholesterol, triglycerides, APOE e4, and fasting status) between individual lipid species and lipid ratios (112 individual species, totaling 12,544 ratios) with ADAS-Cog 13 identified 3385 ratios associated with an uncorrected *p*-value of <0.05 and 1552 significant post-FDR correction (Supplementary Data 1). This analysis confirmed that ratios of short to longer chain sphingomyelins, in particular the ratio of SM(d43:1)/SM(d34:1), presented with a positive association with ADAS-Cog 13 scores (FDR corrected *p*-value of 3.98 × 10^−2^).

### Using the SM species for genetic screening and pathological markers in AD

To link SM readouts associated with AD to genes, we performed metabolite genome-wide association studies (mGWAS) with the three SMs reported to be associated with markers of AD in Toledo et al.^20^, as well as the selected ratio of SM(d43:1)/SM(d34:1). The discovery analysis was performed in a subset of 674 ADNI-1 participants from Toledo et al.^20^ that had genome-wide genotyping data available. While the three single SM species did not yield significant results, the SM ratio was associated with *SPTLC3 (*enzyme *A* in Fig. 1) at genome-wide significance corrected for four metabolic traits (lead SNP rs680379, *p*-value = 1.01 × 10^−9^). This association replicated a previous finding in a larger population-based mGWAS investigating metabolite ratios (rs168622, *r*^*2*^ = 0.98 with rs680379, *p*-value = 5.2 × 10^−25^)^21^.

**Fig. 4:**
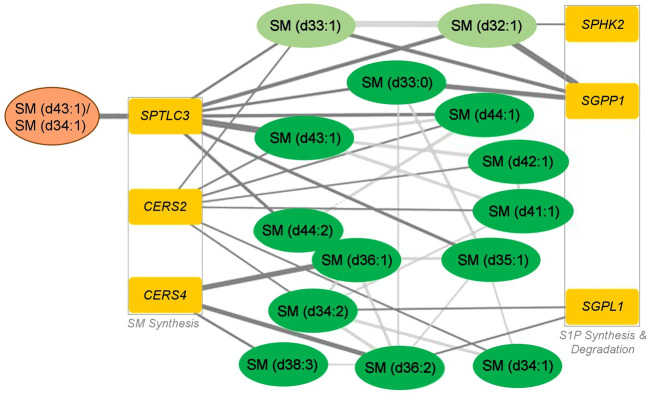
Hybrid network of genetic associations revealed by gene-based association studies and significant partial correlations of detected sphingomyelins^9, 20^. The six identified genes can be grouped into two categories: global sphingomyelin synthesis and synthesis and degradation of sphingosine-1-phosphate. The selected SM ratio is colored orange, other SM species are in green (light green: non-targeted metabolomics in Shin et al.^9^; dark green: targeted metabolomics in ADNI and Draisma et al.^7^), and genes are in dark yellow. S1P Sphingosine-1-phosphate, SM sphingomyelin species.

Lookup of the *SPTLC3* (enzyme *A* in Fig. 1) locus using the large collection of metabolite–genotype associations in the *SNiPA* database^52^) revealed significant links to several additional SM species. To obtain a comprehensive map of genetic influences on SM levels across the whole SM pathway, we again used gene-based association analyses including all 35 genes in the pathway analogously to the analysis of associations with markers of AD. To this end, we used an expanded set of 1407 ADNI participants with SM readouts and genome-wide genotype information available, as well as two large population-based mGWAS studies that included SM levels^7,9^. We found genome-wide and gene-wide significant associations with a set of 14 related SMs for six genes (Fig. 4, Supplementary Table 4). Three of the encoded enzymes are involved in SM synthesis (*SPTLC3, CERS2, CERS4*), while the other three function in the synthesis and degradation of S1P (*SPHK2, SGPP1, SGPL1*), a central exit route of the pathway. Notably, the significant associations include all three SMs identified by Toledo et al.^20^ (SM (d33:0), SM (d34:1), and SM (d38:2)), highlighting a potential role for S1P metabolism and signaling in AD pathogenesis.

### Fingolimod treatment produces a reversal of synaptic plasticity and cognitive impairment in 9-month-old APP/PS1 mice

APP/PS1 are double transgenic mice expressing chimeric amyloid precursor protein (APP) and mutant human presenilin 1 (PS1). These mice are a valuable model to study AD progression and the effect of drugs on AD^53^. To functionally investigate the involvement of deregulated S1P metabolism in amyloid pathology along with strategies to counter AD pathogenesis, we applied a drug repositioning approach by treating amyloidogenic APP/PS1 mice with fingolimod (FTY720), an FDA-approved drug for the use in the relapsing‐remitting form of multiple sclerosis^54^. This immunomodulating compound is a sphingosine analog that, after endogenous phosphorylation by sphingosine kinases 1 and 2, broadly binds to S1P receptors (S1PR1/3/4/5)^55,56^.

It has been previously shown that the first signs of impairment in cognitive performance and synaptic plasticity occur as early as 5 months of age in APP/PS1 mice^57^. We, therefore, decided to establish the onset of fingolimod treatment at 7 months old (mo) for our rescue studies and set out to confirm cognition and synaptic plasticity deficits in APP/PS1 mice compared to WT mice at this age. We tested mice in two behavioral tasks, the novel object recognition (NOR) task, and the Barnes maze (BM) task, to assess episodic and spatial memory, respectively. After behavioral testing, long-term synaptic potentiation (LTP) was evaluated at the hippocampal Schaffer collateral-CA1 (CA3-CA1) synapses and at the lateral entorhinal intracortical synapses (LEC-LEC) to assess synaptic plasticity in the hippocampus and entorhinal cortex, two brain areas compromised in AD^58–62^.

APP/PS1 mice showed a significant deficit in the NOR task compared to WT mice (Supplementary Fig. 2a, unpaired two-sided Student’s *t*-test; *p* = 0.04). APP/PS1 mice showed a mild deficit during the first two training days in the BM task. However, there was no significant difference when comparing the interaction genotype and trial (Supplementary Fig. 2b, two-way repeated measure ANOVA, Šídák’s post hoc; *p* = 0.5). However, during the memory probe trial of the BM task, APP/PS1 mice spent significantly less time in the target/escape hole than their WT littermates (Supplementary Fig. 2c; unpaired two-sided Student’s *t*-test; *p* = 0.02). Consistent with the behavioral findings, APP/PS1 mice showed abnormal LTP expression compared to WT mice at both the CA3-CA1 (Supplementary Fig. 2d and e, unpaired two-sided Student’s *t*-test; *p* = 0.0002) and LECII-LECII (Supplementary Fig. 2f, g, unpaired two-sided Student’s *t*-test; *p* = 0.003).

**Fig. 5:**
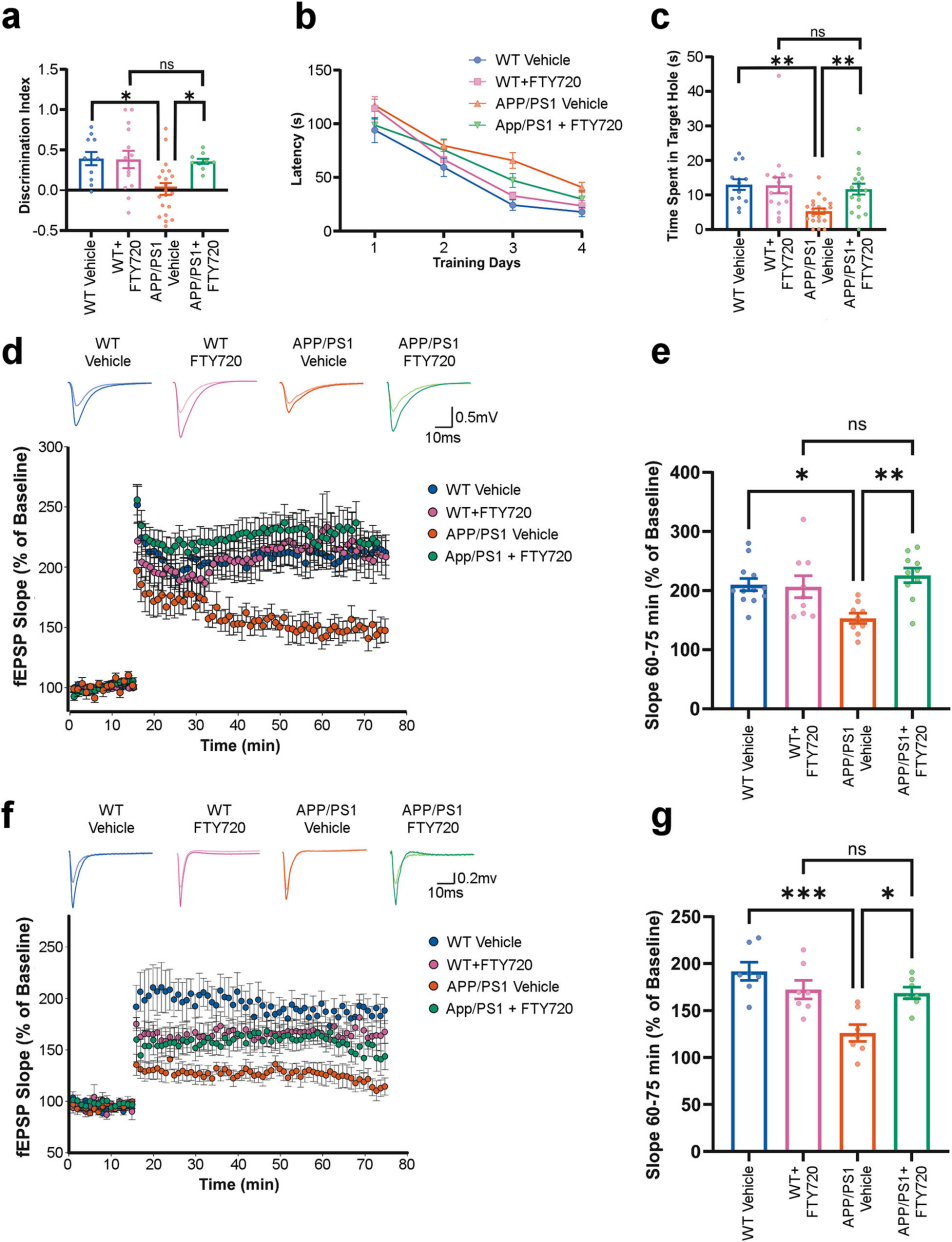
Fingolimod (FTY720) ameliorates memory and synaptic impairment in APP/PS1 mice. **a** Exploration time spent on the novel object in the NOR test session. Data are expressed as a discrimination index ± SEM. FTY720 treatment significantly enhances the discrimination index of the APP/PS1 mice at 9 mo. **b** Barnes maze performance during training days. Acquisition learning trials were performed, and the time it took to locate and enter the escape box is reported in seconds. The average performance of four trials per day is expressed as mean ± SEM. A shorter latency indicates faster spatial learning. No significant difference across trials between APP/PS1 treated and untreated was found. **c** Probe trial was performed on day 5 of the Barnes Maze protocol, during which the escape box was removed. The time spent in the target/escape hole is plotted ±SEM. A larger percentage of time indicates better spatial memory. FTY720 mitigated the spatial learning deficits of the APP/PS1 at 9 mo. **d** CA3 to CA1 synapse LTP. The four small line graphs are representative analog traces of evoked EPSPs before (light colors) and after (dark colors) high-frequency stimulation (HFS). The large plot graph is an LTP timeline. Plotted are normalized evoked excitatory postsynaptic potentials (EPSPs) slopes (*Y*) vs. recording time (*X*). The first 15 min of evoked responses were normalized and used as the baseline responses of LTP. **e** The magnitude of LTP was determined according to the responses between 60 and 75 min after the HFS. Data represent mean fEPSP Slope ± SEM. A rescue of LTP at the CA3-CA1 synapse in APP/PS1 mice at 9 mo is observed after chronic FTY720 treatment. **f** LECII to LECII synapse LTP. The four small line graphs are representative analog traces of evoked EPSPs before (light color) and after (dark color) HFS. The large plot graph is an LTP timeline. **g** LTP magnitude between 60 and 75 min after the HFS. Data represent mean fEPSP Slope ±SEM. FTY720 treatment rescues LTP at the LECII–LECII synapse in APP/PS1 mice at 9 mo. ∗*p* < 0.05, ∗∗*p* < 0.01, and ∗∗∗*p* < 0.001. fEPSP Field excitatory post-synaptic potentials, WT Wild type.

To test the potential reversal effect of fingolimod on cognitive performance and synaptic plasticity, APP/PS1 and WT mice at 7 mo were treated with fingolimod (1 mg/kg/day) for 8 weeks. In the NOR task, fingolimod-treated APP/PS1 mice significantly enhanced their ability to recognize a novel object than the APP/PS1 vehicle group (Fig. 5a, one-way ANOVA, Tukey’s post hoc test; *p* = 0.03; probabilities against chance are shown in Supplementary Table 5). Moreover, APP/PS1-treated mice had similar values in NOR discrimination index as compared to treated WT mice (Fig. 5a, one-way ANOVA, Tukey’s post hoc test; *p* = 0.99). In the BM task, fingolimod-treated APP/PS1 mice also showed better retention memory in the probe trial than the APP/PS1 vehicle group (Fig. 5c, Kruskal–Wallis, Dunn’s post hoc; *p* = 0.004; probabilities against chance are shown in Supplementary Table 5), and no statistical difference was observed between the APP/PS1-treated compared to the WT-treated group (Fig. 5c, Kruskal–Wallis, Dunn’s post hoc; *p* = 0.99). Analysis of training performance in the BM task showed no significant interaction between treatment effects and genotype across days (Fig. 5b, two-way repeated measure ANOVA, Tukey’s post hoc; *p* = 0.3). These findings suggest that fingolimod effectively enhanced both episodic and spatial memory in an amyloidogenic AD mice model.

To confirm the mechanistic underpinnings of this behavioral rescue, we tested the effect of fingolimod on synaptic plasticity. Fingolimod treatment significantly augmented LTP expression in APP/PS1 mice at the CA3–CA1 (Fig. 5d) and LECII–LECII (Fig. 5f) synapses. LTP expression in fingolimod-treated APP/PS1 and WT mice was indistinguishable at the CA3–CA1 synapse (Fig. 5e, two-way ANOVA, Tukey’s post hoc; *p* = 0.7) and the LECII–LECII synapse (Fig. 5g, two-way ANOVA, Tukey’s post hoc; *p* = 0.99). These data indicate that S1P pathway modulation via prolonged fingolimod treatment can rescue cellular and cognitive deficits in the APP/PS1 mouse model of AD.

## Discussion

This study systematically analyzed the SM pathway for multi-omics links to pathogenic processes in AD. We were able to replicate the findings in human post-mortem samples, in vivo samples, and mouse models. The key findings from the multiple lines of evidence presented here are: (a) using post-mortem brain transcriptome data of 2114 samples, we identified differentially expressed genes in the SM pathway of AD patients; (b) comparison of 1708 context-specific metabolic reconstruction of the brain regions showed differences in the reaction fluxes for AD and NCI samples; (c) multimodal neuroimaging analysis of 1576 individuals identified genetic variants linked to genes in SM pathway and associated with AD pathogenesis; (d) plasma metabolomic and lipidomic analysis identified the SM(d43:1)/SM(d34:1) ratio as a strong intermediate trait for sphingolipid dysregulation in AD; (e) metabolite genome-wide association studies (mGWAS) identified S1P metabolite as potential AD drug target; and (f) experimental analyses of amyloidogenic APP/PS1 mice treated with fingolimod revealed beneficial effects of S1P modulation and alleviated synaptic plasticity and cognitive impairment in mice.

We demonstrated that, on the gene expression level, the SM pathway is globally dysregulated across brain regions in samples of AD cases compared to controls. We found that 20 out of 35 genes encoding the core enzymes in the pathway are significantly differentially expressed in the AD population. The only sub-pathway that appears to be unaffected by or uninvolved in the disease is the synthesis and recycling of glycosphingolipids. Using constraint-based metabolic networks of brain regions integrated with post-mortem brain transcriptome data, we further show that the differential expression of the enzymes involved in at least three reactions is predicted to result in significant flux differences in AD cases versus controls. An increase in the flux for the reaction catalyzed by serine palmitoyl transferase (SPT) between AD, control, and MCI was consistent with the expression level of *SPT* gene in these groups. It was interesting to observe a higher flux distribution for MCI group. Some of the previous studies have shown that level and range of hypermethylation are relatively higher in MCI than in AD cases^63–65^. Hypermethylation in the promoter region has been studied with respect to the upregulation of gene expression and it might have important implications. It will be interesting to analyze the methylation state of the promoter region of the SPT gene in MCI and AD cases to support our hypothesis. We also observe an increased flux of reaction catalyzed by sphingomyelin synthase (SGMS*)*. Studies have shown that elevated SGMS activity and sphingomyelin levels impacted APP processing to produce Aβ and are a potential contributing factor in Aβ pathology associated with AD^66–68^. While flux differences cannot be directly interpreted with respect to the resulting metabolic changes, there is ample evidence from metabolomics studies that the pathway exhibits differential output in AD.

We next assessed the association of genes in the SM pathway with A-T-N-C measures of AD by investigating genetic associations of CSF biomarker levels, brain atrophy (magnetic resonance imaging), brain glucose metabolism ([^18^F] FDG-PET), cognition, and clinical diagnosis. Ten of the 35 genes in the pathway showed significant associations with at least one (endo)phenotype at the gene level. Although not genome-wide significant, this large coverage of genes in the SM pathway suggests that there might be at least a small fraction of genetic risk predisposition to AD attributable to the pathway as a whole. Using SM levels as intermediate traits for the genetic association, screening further revealed six central enzymes in the pathway to be genetically influencing levels of a network of 14 SM species. As all the genetic variants associated with SM levels were linked to the respective enzymes via expression quantitative trait loci, this indicates that some of the genetic links between the pathway and markers of AD may be mediated by altered regulation of SM levels via genetically influenced differential gene regulation.

While associations from the analysis of differential gene expression in brain tissue as well as from the phenotype GWASs were broad and generally implicated in SM pathway function, the associations from the SM mGWASs linked two central pathway routes: global SM synthesis and S1P metabolism. Based on previous mGWAS analysis, genetic associations with core enzymes involved in the primary synthesis of SM metabolites are expected. However, the specific association with one particular exit route out of the pathway (via sphingosine and S1P) is striking. Five of the six detected genes (*SPTLC3, CERS2, CERS4, SPHK2*, and *SGPL1*) were also found to be significantly linked to AD either through differential gene expression or via genetic associations or both, which suggests that S1P metabolism may be relevant to disease.

S1P is known to be involved in endothelial barrier function in a context-dependent manner. Decreased S1P by lipopolysaccharide (LPS) treatment produced blood–brain barrier (BBB) abnormalities, and increased activity of SGPP1 and S1PR^28^. Chronic BBB leakiness is associated with cognitive impairment, but not with signs of brain inflammation^29^. S1P in general increases neuronal and circuit excitability^30,31^. Depletion of the S1P-producing enzyme SphK1 induces an impairment of mossy fiber—CA3 LTP and deficits in spatial reference memory^32^. Depletion of SphK2 produced lower levels of hippocampal S1P, reduced histone acetylation and deficits in spatial memory as well as impaired contextual fear extinction^33^. Thus, S1P, SphK1, and SphK2 play specific roles in brain areas serving specific memory functions through intracellular S1P effects as well as signaling pathways downstream of S1P GPCRs. A recent study showed that Aβ1–42 enhanced SphK1 expression and activity after 24 h, but down-regulated them after 96 h and had no effect on Sphk2. Aβ1–42 and SKI II-induced free radical formation, disturbed the balance between pro- and anti-apoptotic proteins and evoked cell death in PC12 cells while SP1 rescued part of this damage^37^. S1P may act as a second messenger, but it can also be transported to extracellular space and may affect cell function via stimulation of the receptors (S1PR1–5). Two modulators of SP1R1 (Fingolimod and SEW2871) have been shown to improve Aβ-mediated behavior abnormalities and decrease tau phosphorylation.

To explore the effect of fingolimod administration on cognition and plasticity, we used the APP/PS1 mouse model for AD. Fingolimod is a sphingosine-1-phosphate receptor modulator approved for treating multiple sclerosis in the US^54^. APP/PS1 mice had a significant deficit in cognitive and memory behavior and synaptic function that was reversible with FTY720 treatment. These results suggest that fingolimod modulates the S1P pathway to alleviate AD-associated deficits in APP/PS1 mice. The effect of fingolimod in APP/PS1 on behavior and synaptic transmission can be direct or through the activation of S1P receptors or both since they are not mutually exclusive.

To date, despite its potential therapeutic relevance, fingolimod research in mice models of AD is scarce. Two studies using 5xFAD female mice found that fingolimod treatment halted spatial memory decline assessed in a Morris Water Maze (MWM) task and expression of pathological biomarkers^69,70^. Another study found changes in gene expression profiling in the brains of FVB-Tg females after two weeks of FTY720 treatment^71^. Lastly, a reversal treatment study found that 8-week fingolimod treatment recovered deficits in dendritic spines, CA3–CA1 synaptic plasticity, and spatial memory in an MWM task in eight months old APP/PS1 males^72^. Our results extend previous observations on the positive effects of fingolimod treatment on AD mouse models. They also expand the scope of the reversal treatment to more AD-relevant cognitive tasks and synaptic circuits. We examined older (9 months old), more compromised animals of both sexes. Our choice of behavioral tasks, namely NOR and Barnes maze, differs from MWM, being both less stressful and driven by exploratory behavior on novelty and sheltering and distinguishing episodic and spatial memory, respectively. In addition, our examination of synaptic function targeted hippocampal and entorhinal cortex circuits, two areas that are among the first neural systems affected in AD^58–62^, and coordinate Barnes and NOR task performance.

This study integrated multi-omics analyses from AD patients and led to an experimental strategy to use an animal model to identify multiple, dysregulated steps in SM metabolism. It provides a link between SM dysregulation and changes in brain function. The approach used here opens the possibility of repurposing fingolimod, or other S1P modulators, for the treatment of AD. Fingolimod has been shown to modulate both amyloid and tau pathology in AD models^70,71,73^ and it has been proposed to be neuroprotective by modulating S1P signaling in the brain^74^. A recent study used network pharmacology methods and showed the probable pharmacological mechanism of fingolimod in the frontal cortex region of AD patients^75^. Rescuing both synaptic and cognitive function (cellular substrate and behavioral end-result) with fingolimod is a compelling finding, which provides evidence for dysregulated S1P signaling in AD mice and further supports the identification of this pathway as a high-priority candidate AD drug target.

## Methods

### Identification of differential gene expression in brain tissue RNA-seq data

We used the reprocessed AMP-AD RNA-seq data available from three studies—the Religious Order Study and the Rush Memory and Aging Project (ROS/MAP), the Mount Sinai Brain Bank cohort (MSBB), and the Mayo clinic RNA-seq study^43^— covering seven brain regions (cerebellum, temporal cortex, dorsolateral prefrontal cortex, parahippocampal gyrus, frontal pole, inferior frontal gyrus, and superior temporal gyrus), as well as a published meta-analysis of these datasets^43^ to identify genes in the SM pathway that are differentially expressed in AD cases compared to controls. Gene expression changes were considered significant at an FDR-corrected*p*-value ≤ 0.05. All datasets are publicly available, see the “Data availability” Statement. The data used for the analyses described in this manuscript were obtained from the GTEx Portal on 02/10/22.

### In silico metabolic flux analysis using brain region-specific metabolic networks

Genome-scale metabolic networks of brain regions were reconstructed in our previous study^76^ and we used these metabolic networks for our present work. We integrated the post-mortem brain transcriptome data as mentioned in ref. ^76^. Using iMAT algorithm^77^, we generated context-specific personalized metabolic networks for each post-mortem sample in the dataset. Human cells in general do not proliferate rapidly and they tend to maintain their metabolic functions^78^. We, therefore, chose the biomass maintenance reaction, glutamate, and glutamine exchange as the objective function for the brain regions. We used dorsolateral prefrontal cortex samples for the present analysis. We performed flux variability analysis (FVA) to evaluate minimum and maximum flux for each reaction in the metabolic networks. We carried out the analysis for all context-specific metabolic networks. We selected the reactions that were part of sphingolipid metabolism using the subsystem definition. We used the *scale* function in R to normalize the flux values and applied the Wilcoxon test (rstatix package) to the normalized values to identify reactions with an adjusted *p*-value of <0.05 in AD versus NCI and MCI samples. These reactions were identified as significant reactions in the groups. We used COBRA toolbox v3.0^79^ for metabolic analysis that was implemented in MATLAB R2018a and academic licenses of Gurobi optimizer v7.5 and IBM CPLEX v12.7.1 were used to solve LP and MILP problems.

### Neuroimaging processing and analysis

Participants of the Alzheimer’s Disease Neuroimaging Initiative (ADNI) were used in the analysis. Demographic information, imaging scan data, neuropsychological test scores, and clinical information were downloaded from the ADNI data repository (www.loni.usc.edu). As described in detail in previous studies^80,81^, T1-weighted structural magnetic resonance imaging (MRI) scans were processed by using a widely employed automated MRI analysis technique (FreeSurfer) to extract cortical thickness. Pre-processed [^18^F] FDG positron emission tomography (PET) scans were downloaded. Methods for the acquisition and processing of PET scans were described previously^80^. [^18^F] FDG-PET scans were intensity-normalized using a pons region of interest to create standardized uptake value ratio (SUVR) images. For surface-based whole brain analysis of cortical thickness on a vertex-by-vertex basis, the SurfStat software package (www.math.mcgill.ca/keith/surfstat/) was used to perform a multivariable analysis of generalized linear regression to examine the association of genetic variation on brain structural changes. Age, sex, years of education, intracranial volume, and magnetic field strength were used as covariates. In order to adjust for multiple comparisons, the random field theory correction method was used with *p* < 0.05 adjusted as the level for significance. For whole-brain analysis of brain glucose metabolism on a voxel-wise basis using the processed FDG-PET images, SPM12 (www.fil.ion.ucl.ac.uk/spm/) was used to investigate the effect of genetic variation on brain glucose metabolism across the whole brain. Age and sex were used as covariates. In order to adjust for multiple comparisons, the significant statistical parameters were selected to correspond to a threshold of *p*-value < 0.05 (FDR-corrected).

### Assessment of SM ratios using targeted metabolomics in ADNI-1

For the investigation of SM ratios measured by targeted metabolomics using the Biocrates P180 kit, we used the same cohort data and statistical models used in Toledo et al.^20^. For the selection of the most informative SM ratio, we first calculated all ratios between short-chain (chain length < C20) and long-chain (≥C20) SMs on metabolite levels not adjusted for medication. For each ratio, we then identified significant medications using backward selection based on the Bayesian Information Criterion. Significant medications were included as additional covariates extending the base models described in Toledo et al.^20^ for phenotype associations. Using the Pgain criterion, which is defined by the ratio of the minimum association *p*-value of the constituents of a ratio with the association *p*-value of the ratio and provides a measure of significance added by the ratio, we obtained the ratio of SM (d34:1) and SM (d43:1) as the one with the largest overall Pgain.

### Replication analysis of SM ratios using targeted lipidomics in ADNI-1

A more detailed lipidomics method was applied in the ADNI-1 samples to obtain better coverage of the sphingolipidome. Methodology on the ADNI cohort was as described by Huynh et al.^21^. In brief, extracted samples were run using reverse phase liquid chromatography coupled with a triple quadrupole mass spectrometer (Agilent 6490, Agilent). Characterization of sphingolipid isomers has been reported previously^82^ where repeated pooled runs using differing mass spectrometry conditions to obtain structurally informative fragments in MS/MS. Ratios were generated using 112 sphingolipid species and log2-transformed. Linear regression with ADAS-Cog. 13 was done with age, sex, BMI, HDL-C, total cholesterol, clinical triglycerides, fasting status, and APOE e4 genotype as covariates. *p*-values were corrected for multiple correction comparison using the Benjamini and Hochberg approach^83^.

### Candidate mGWAS analysis in ADNI-1

We downloaded genome-wide genotype data for ADNI-1 participants from LONI. Genotype quality control (QC) included the exclusion of samples and genotypes with <95% call rate and exclusion of variants that violated a Hardy–Weinberg equilibrium (HWE) test *p*-value of 1 × 10^−5^ or had a minor allele frequency (MAF) < 5%. We then performed autosomal mGWAS analysis with the three SMs (SM (d32:0), SM d(34:1), SM (d38:3)) reported as significantly associated with markers of AD in Toledo et al.^20^, as well as the SM(d43:1)/SM(d34:1) ratio reported here. As covariates, we included age, sex, diagnostic group, as well as the first five components derived by multidimensional scaling (MDS) analysis to account for population stratification. The threshold for genome-wide significance adjusted for four metabolic traits was *p*-value ≤ 1.25 × 10^−8^. Genetic associations were calculated using PLINK v1.9^84^.

### Phenotype GWAS and global SM mGWAS analysis in ADNI-1/GO/2

Genome-wide genotyping data of ADNI-1/GO/2 participants were collected using the Illumina Human 610-Quad, HumanOmni Express, and HumanOmni 2.5 M BeadChips. Before imputation, standard QC procedures of GWAS data for genetic markers and subjects were performed (variant call rate <95%, HWE test *p*-value <1 × 10^−6^, and MAF < 1%, participant call rate < 95%, sex check and identity check for related relatives). Then, non-Hispanic Caucasian participants were selected using HapMap 3 genotype data and MDS analysis. Genotype imputation was performed for each genotyping platform separately using the Haplotype Reference Consortium (HRC) reference Panel r1.1 and merged afterward, resulting in data on 1576 individuals and 20,779,509 variants. Using this dataset, we ran GWAS analyses for each outcome (A-T-N-C measures, clinical diagnosis, and metabolite levels) that included outcome-specific sets of covariates. These are listed in Supplementary Table 6, along with the respective numbers of included individuals and genetic variants.

### Annotation of genetic variants and gene-wide significance thresholds

Previously reported metabolite associations for genes in the SM pathway were retrieved from SNiPA^52^, which was also used to identify overlapping expression quantitative trait loci (eQTLs) from multiple sources. Effect directions of genotype–metabolite and eQTL associations were obtained from the original publications^52^. SNiPA was also used to project genetic variants to genes, a process that includes mapping of variants to genes via genomic location links to genes via expression and protein QTLs, as well as a location in a gene-associated promoter or enhancer region^52^. The number of all genetic variants projected to a particular gene was used to derive gene-wise Bonferroni thresholds for significant genetic associations (*p*-value ≤ 0.05/(number of variants)). Furthermore, as SNPs within genes are correlated due to linkage disequilibrium and Bonferroni correction is often too conservative, we used the permutation test, which provides a gene-based empirical *p*-value that corrects for the number of SNPs within each gene by randomly permuting the phenotypes multi times (20,000 times) and performing statistical tests for all permuted data sets.

### Mouse model

Experiments were approved by the Division of Comparative Medicine (DCM) from SUNY Downstate Medical Center. APPswe/PS1dE9 (referred to as APP/PS1) and C57Bl/6J (referred to as WT) mice were purchased from The Jackson Laboratory. The APP/PS1 is a double transgenic mouse expressing a chimeric mouse/human amyloid precursor protein (Mo/HuAPP695swe) and a mutant human presenilin 1 (PS1-dE9) both directed to central nervous system neurons^85^. For all behavioral and synaptic studies at 7 and 9 mo, each experimental group comprised both males and females (Supplementary Table 5). We found no significant differences due to sex within each experimental group (data not shown).

### Fingolimod administration

To determine if fingolimod oral administration achieves appropriate plasma concentration, we treated 8 WT mice at 7 mo (50% Females) with fingolimod at 1 mg/kg/day for 4 wks. Plasma samples were collected at two-time points (2nd and 4th weeks) after treatment and analyzed by UHPLC and MS–MS. Fingolimod levels in plasma were in ng/ml: 2nd week = 8.03 ± 0.24 and 4th week = 10.02 ± 0.4. The results indicate that oral administration is an appropriate route for mice experiments.

We used APP/PS1 and their wild-type littermates to examine fingolimod effects in vivo. Fingolimod treatment was provided in drinking water in a dark container and changed every 48 h to provide 1 mg/kg/day.

### In vitro electrophysiological recordings

Mice were anesthetized with Ketamine/Xylazine (100/10 mg/kg) and decapitated with an animal guillotine. Horizontal hippocampal slices (400 μm) were prepared using a Vibrotome slicer (VT 1000S; Leica) in ice-cold cutting solution containing the following in mM: 130 potassium gluconate, 5 KCl, 20 HEPES acid, 25 glucose, 0.05 kynurenic acid, 0.05 EGTA-K, and pH equilibrated at 7.4 with KOH. After slicing, the tissue was allowed to recover for an hour before the beginning of experiments in artificial CSF (aCSF) that contained the following in mM: 157 Na^+^, 136 Cl^−^, 2.5 K^+^, 1.6 Mg^2+^, 2 Ca^2+^, 26 HCO_3_^−^, and 11 d-glucose.

LTP recordings were performed in an interface chamber (Fine Scientific Tools, Vancouver, Canada) and slices were perfused with aCSF continuously bubbled with 95% O_2_/5% CO_2_, to maintain pH near 7.4 and the temperature was set at 34 °C. Field excitatory post-synaptic potentials (fEPSPs) were recorded in the CA1 stratum radiatum and lateral entorhinal cortex superficial layer II (LECII) with a glass electrode filled with aCSF (2–3 MΩ resistance), and the fEPSPs were elicited by stimulating the Schaffer collateral fibers and LECII with a bipolar electrode. Input–output curves were obtained, and a stimulus that evoked ~40% of the maximum fEPSP was selected to record the baseline. Baseline responses were obtained (15 min with an inter-stimulus interval of 20 s) before high-frequency stimulation (HFS) one train of 100 stimuli at 100 Hz and three trains of 100 stumuli at 100 Hz, with 10 s intervals were used to induce synaptic LTP at the CA3–CA1 and LECII–LECII synapses, respectively. Responses were recorded for 60 min after HFS. The tungsten stimulating electrodes were connected to a stimulus isolation unit (Grass S88), and the recordings were made using an Axoclamp 2B amplifier (Molecular Devices) and then filtered (0.1 Hz–10 kHz using −6 dB/octave). The voltage signals were digitized and stored on a PC using a DigiData 1200A (Molecular Devices) for offline analysis. The fEPSP slope was measured and expressed as a percentage of the baseline. The data was analyzed using Axon™ pCLAMP™ software, and the results are expressed as the mean ± standard error of the mean (SEM). Data were analyzed statistically using two-way ANOVA or an Unpaired two-sided Student’s *t*-test, as described in the figure legend, with the GraphPad Prism package.

### Novel object recognition (NOR)

Mice were habituated to experimental apparatus consisting of a gray rectangular open field (60 cm × 50 cm × 26 cm) for 5 min in the absence of any objects for 3 days. On the third day, after the habituation trial, mice were placed in the experimental apparatus in the presence of two identical objects and allowed to explore them for 5 min. After a retention interval of 24 h, mice were placed again in the apparatus, where one of the objects was replaced by a novel object. All sessions were recorded using Noldus Media Recorder software. Exploration of the objects was defined as the mice facing and sniffing the objects within 2 cm distance and/or touching them, assessed with ANY-maze software. The ability of the mouse to recognize the novel object (discrimination index) was determined by dividing the difference between exploration time devoted to the novel object and the time devoted to the familiar object by the total time exploring the novel and familiar objects during the test session. For the comparison of WT and APP/PS1 treated and untreated performance in the NOR task, a one-way ANOVA with a Tukey’s post hoc was used. An Unpaired two-sided Student’s *t*-test was used to compare WT and APP/PS1 at 7 mo. All the statistical analyses were performed using GraphPad Prism version 9.00 for Windows (GraphPad Software).

### Barnes maze

The behavioral apparatus consisted of a white flat, circular disk with 20 holes around its perimeter. One hole held the entrance to a darkened escape box not visible from the surface of the board, allowing the subject to exit the maze. The escape chamber position remained fixed during all trials. Mice learn the location of the escape hole using spatial reference points that were fixed in relation to the maze (extra-maze cues). The task consisted of one habituation trial on day 1 where the escape hole was presented to the animal, the animal remained in the escape box for 2 min. After the habituation trial, the training phase consisted of four 3-min trials of spatial acquisition for 4 consecutive days with a 15 min inter-trial interval. On the fifth day (probe trial) the escape box was removed, and the animals were allowed to explore the maze for 90 s. All sessions were recorded using Debut video software and assessed through ANY-maze software. For each trial, several parameters were recorded to assess performance. These include the latency to locate the escape box, the number of incorrect holes checked prior to entering the escape box, as well as the distance traveled prior to locating the escape box. For the probe trial, the time spent on the target hole was analyzed. A two-way repeated measure ANOVA was applied to compare escape latency across days between groups. For the comparison of WT and APP/PS1 treated and untreated performance in the probe trial, a Kruskal Wallis test with a Dunn’s post hoc was used. An Unpaired two-sided Student’s *t*-test was used to compare WT and APP/PS1 at 7mo. All the statistical analyses were performed using GraphPad Prism version 9.00 for Windows (GraphPad Software).

### Statistics and reproducibility

Statistical analyses of gene expression and reaction fluxes were done in R using the *rstatix package*. The genome-scale metabolic networks were analyzed using COBRA toolbox v3.0^79^ implemented in MATLAB R2018a. Academic licenses of Gurobi optimizer and IBM CPLEX v12.7.1 solvers were used in MATLAB. SurfStat software package (www.math.mcgill.ca/keith/surfstat/) was used to perform a multivariable analysis of generalized linear regression to examine the association of genetic variation on brain structural changes in the neuroimaging data. For the lipidomics analysis, we used age, sex, diagnostic group, and the first five components derived by multidimensional scaling for population stratification and used PLINK v1.9^84^ for calculating genetic associations. All the statistical analyses in APP/PS1 and WT mice were performed using the GraphPad Prism version 9.00 for Windows (GraphPad Software).

### Reporting summary

Further information on research design is available in the Nature Research Reporting Summary linked to this article.

## Supplementary information

Supplementary Information

Description of Additional Supplementary Files

Supplementary Data 1

Reporting Summary

## Data Availability

Metabolomics datasets from the AbsoluteIDQ-p180 metabolomics kit used in the current analyses for the ADNI-1 and ADNI-GO/−2 cohorts as well as the RNASeq data from the ROS/MAP, Mount Sinai Brain Bank Cohort, and the Mayo Clinic cohort are available via the Accelerating Medicines Partnership-Alzheimer’s Disease (AMP-AD) Knowledge Portal and can be accessed at10.7303/syn5592519 (ADNI-1), 10.7303/syn9705278 (ADNI-GO/−2), 10.7303/syn3388564 (ROS/MAP),10.7303/syn3157743 (MSSB), and 10.7303/syn5550404 (Mayo clinic). The source data behind the graph in Fig. 2 can be found in https://figshare.com/articles/dataset/merged_file_reactions_Maxflux/20769229 and https://figshare.com/articles/dataset/Covariate_file/20769232. The full complement of clinical and demographic data for the ADNI cohorts are hosted on the LONI data sharing platform and can be requested at http://adni.loni.usc.edu/data-samples/access-data/. The full complement of clinical and demographic data for the ROS/MAP cohorts is available via the Rush AD Center Resource Sharing Hub and can be requested at https://www.radc.rush.edu.
