# Supplementary material for: Multi-Omic analyses characterize the ceramide/sphingomyelin pathway as a therapeutic target in Alzheimer’s disease
Source: Commun Biol. 2022 Oct 8;5:1074. doi: 10.1038/s42003-022-04011-6 (PMC9547905; doi:10.1038/s42003-022-04011-6)
Supplement: Supplementary file 1 — Supplementary Information [file 42003_2022_4011_MOESM1_ESM.pdf]

Supplementary Information

Multi-Omic Analyses Characterize the Ceramide/Sphingomyelin Pathway as a Therapeutic Target in Alzheimer's Disease

Priyanka Baloni[1,2]*, Matthias Arnold[3,4]*, Luna Buitrago[5*], Kwangsik Nho[6]*, Herman Moreno[5], Kevin Huynh[7], Barbara Brauner[3], Gregory Louie[4], Alexandra Kueider-Paisley[4], Karsten Suhre[8], Andrew J. Saykin[6], Kim Ekroos[9], Peter J. Meikle[7], Leroy Hood[1], Nathan D. Price[1], The Alzheimer's Disease Metabolomics Consortium, P. Murali Doraiswamy[4], Cory C. Funk[1], A. Iván Hernández[10], Gabi Kastenmüller[3], Rebecca Baillie[11], Xianlin Han[12‡], and Rima Kaddurah-Daouk[4,13,14]. A full list of The Alzheimer's Disease Metabolomics Consortium members appears at the end of this paper.

* Equal contribution

Author's affiliations

[1] Institute for Systems Biology, Seattle, WA, USA

[2] School of Health Sciences and Purdue Institute for Integrative Neuroscience, Purdue University, West Lafayette, IN, USA

[3] Institute of Computational Biology, Helmholtz Zentrum München - German Research Center for Environmental Health, Neuherberg, Germany

[4] Department of Psychiatry and Behavioral Sciences, Duke University School of Medicine, Durham, Durham, NC, USA.

[5] Department of Neurology/Pharmacology, SUNY Downstate Medical Center, Brooklyn, NY, USA

[6] Indiana Alzheimer's Disease Research Center and Department of Radiology and Imaging Sciences, Indiana University School of Medicine, Indianapolis, IN, USA

[7] Metabolomics Laboratory, Baker Heart and Diabetes Institute, Melbourne, VIC, Australia

[8] Department of Physiology and Biophysics, Weill Cornell Medicine-Qatar, Education City, PO 24144, Doha, Qatar

[9] Lipidomics Consulting Ltd., Esbo, Finland

[10] Department of Pathology, SUNY Downstate Medical Center, Brooklyn, NY, USA

[11] Rosa & Co LLC, San Carlos, CA, USA

[12] University of Texas Health Science Center at San Antonio, San Antonio, TX, USA

[13] Department of Medicine, Duke University, Durham, NC, USA

[14] Duke Institute of Brain Sciences, Duke University, Durham, NC, USA

Corresponding Authors:

Rima Kaddurah-Daouk, PhD
Email: kaddu001@mc.duke.edu

Xianlin Han, PhD
Email: hanx@uthscsa.edu

Supplementary Table 1. List of enzymes in the sphingomyelin pathway. The list of enzymes was curated from human genome-scale metabolic reconstruction, Recon 3D.

Gene Symbol	Ensembl Gene ID	Gene Name	Reaction	Differential Expression	Trait GWAS	mGWAS
SPTLC1	ENSG00000090054	serine palmitoyltransferase, long chain base subunit 1	serine palmitoyltransferase	x		
SPTLC2	ENSG00000100596	serine palmitoyltransferase, long chain base subunit 2				
SPTLC3	ENSG00000172296	serine palmitoyltransferase, long chain base subunit 3		x	x	x
KDSR	ENSG00000119537	3-ketodihydrosphingosine reductase	3-ketodihydrosphingosine reductase	x		
CERS1	ENSG00000223802	ceramide synthase 1	ceramide synthase	x		
CERS2	ENSG00000143418	ceramide synthase 2		x	x	x
CERS3	ENSG00000154227	ceramide synthase 3			x	
CERS4	ENSG00000090661	ceramide synthase 4		x		x
CERS5	ENSG00000139624	ceramide synthase 5		x		
CERS6	ENSG00000172292	ceramide synthase 6		x	x	
DEGS1	ENSG00000143753	delta(4)-desaturase, sphingolipid 1	dihydroceramide desaturase	x	x	
DEGS2	ENSG00000168350	delta(4)-desaturase, sphingolipid 2				
ACER1	ENSG00000167769	alkaline ceramidase 1	ceramidase			
ACER2	ENSG00000177076	alkaline ceramidase 2		x	x	
ACER3	ENSG00000078124	alkaline ceramidase 3		x		
ASAH1	ENSG00000104763	N-acylsphingosine amidohydrolase (acid ceramidase) 1				
ASAH2	ENSG00000188611	N-acylsphingosine amidohydrolase (non-lysosomal ceramidase) 2				
SGMS1	ENSG00000198964	sphingomyelin synthase 1	sphingomyelin synthase	x	x	
SGMS2	ENSG00000164023	sphingomyelin synthase 2				
SMPD1	ENSG00000166311	sphingomyelin phosphodiesterase 1, acid lysosomal	sphingomyelin phosphodiesterase			

SMPD2	ENSG00000135587	sphingomyelin phosphodiesterase 2, neutral membrane			x	
SMPD3	ENSG00000103056	sphingomyelin phosphodiesterase 3, neutral membrane		x		
SMPD4	ENSG00000136699	sphingomyelin phosphodiesterase 4, neutral membrane		x		
ENPP7	ENSG00000182156	ectonucleotide pyrophosphatase/phosphodiesterase 7				
CERK	ENSG00000100422	ceramide kinase	ceramide kinase	x		
UGCG	ENSG00000148154	UDP-glucose ceramide glucosyltransferase	ceramide glucosyltransferase			
GBA	ENSG00000177628	glucosidase, beta, acid	glucosylceramidase			
SPHK1	ENSG00000176170	sphingosine kinase 1	sphingosine kinase	x		
SPHK2	ENSG00000063176	sphingosine kinase 2			x	x
PLPP1	ENSG00000067113	phospholipid phosphatase 1	phospholipid phosphatase	x		
PLPP2	ENSG00000141934	phospholipid phosphatase 2			x	
PLPP3	ENSG00000162407	phospholipid phosphatase 3		x		
SGPP1	ENSG00000126821	sphingosine-1-phosphate phosphatase 1				x
SGPP2	ENSG00000163082	sphingosine-1-phosphate phosphatase 2		x		
SGPL1	ENSG00000166224	sphingosine-1-phosphate lyase 1	sphingosine-1-phosphate lyase 1	x		x

Supplementary Table 2. Differential expression for genes in the SM pathway. The expression changes were analyzed from post-mortem brain samples and compared in AD vs controls. Statistical analysis of SMS, SPT, and CERK reactions.

Gene Symbol	Ensembl Gene Id	Brain Region- & Study-Specific Analysis				Fixed-Effects Meta-Analysis			Random-Effects Meta-Analysis			Heterogeneity Statistics			
		Log (FC)	q-value	Brain Region	Study	Esti-mate	Std. Error	q-value	Esti-mate	Std. Error	q-value	Q	Tau	H	I2
CERS6	ENSG00000172292	0.894	**1.65E-08**	CBE	MayoRNAseq	0.031	0.066	7.25E-01	0.024	0.189	9.43E-01	47.064	0.466	2.801	0.873
		-0.17	**1.96E-02**	PHG	MSBB										
ACER2	ENSG00000177076	0.498	**4.00E-04**	CBE	MayoRNAseq	0.101	0.066	2.00E-01	0.077	0.125	6.97E-01	20.735	0.280	1.859	0.711
		0.288	**4.32E-02**	TCX	MayoRNAseq										
SPTLC1	ENSG00000090054	0.227	**7.00E-04**	CBE	MayoRNAseq	0.256	0.066	**5.06E-04**	0.261	0.073	**3.13E-03**	6.452	0.084	1.037	0.070
		0.069	6.79E-02	PHG	MSBB										
		0.054	3.42E-01	IFG	MSBB										
		0.203	**2.50E-03**	TCX	MayoRNAseq										
		0.021	7.57E-01	FP	MSBB										
		0.059	2.32E-01	STG	MSBB										
		0.013	8.88E-01	DLPFC	ROSMAP										
SGPP2	ENSG00000163082	0.369	**8.00E-04**	CBE	MayoRNAseq	0.102	0.065	1.94E-01	0.098	0.105	5.29E-01	14.998	0.214	1.581	0.600
CERS4	ENSG00000090661	0.086	1.82E-01	STG	MSBB	0.393	0.066	**8.93E-08**	0.393	0.066	**2.30E-07**	1.532	0	1	0
		0.062	4.12E-01	IFG	MSBB										
		0.181	**6.00E-04**	TCX	MayoRNAseq										
		0.098	**4.68E-02**	PHG	MSBB										
		0.122	**3.27E-02**	FP	MSBB										
		0.155	**4.00E-03**	CBE	MayoRNAseq										
		0.092	**2.87E-02**	DLPFC	ROSMAP										
SMPD4	ENSG00000136699	0.038	4.76E-01	FP	MSBB	0.224	0.066	**2.46E-03**	0.205	0.095	9.42E-02	12.279	0.178	1.431	0.511
		-0.012	8.52E-01	TCX	MayoRNAseq										
		0.014	8.41E-01	IFG	MSBB										
		0.074	**5.40E-03**	DLPFC	ROSMAP										
		0.15	**5.10E-03**	CBE	MayoRNAseq										

		0.034	4.97E-01	STG	MSBB									
		0.004	9.26E-01	PHG	MSBB									
PLPP1	ENSG00000067113	0.177	**7.20E-03**	DLPFC	ROSMAP	0.463	0.066	**3.51E-10**	0.463	0.087	**4.20E-06**	10.079	0.145	1.296	0.405
		0.182	6.90E-02	IFG	MSBB									
		0.157	8.39E-02	STG	MSBB									
		0.247	**4.00E-04**	PHG	MSBB									
		0.19	**6.40E-03**	CBE	MayoRNAseq									
		0.061	5.90E-01	FP	MSBB									
		0.35	**3.10E-07**	TCX	MayoRNAseq									
CERS5	ENSG00000139624	-0.188	**1.01E-02**	CBE	MayoRNAseq	-0.107	0.065	1.71E-01	-0.106	0.066	2.31E-01	6.071	0.018	1.006	0.012
SGPL1	ENSG00000166224	0.089	1.87E-01	IFG	MSBB	0.236	0.066	**1.50E-03**	0.257	0.165	2.50E-01	37.353	0.397	2.495	0.839
		0.189	**6.00E-04**	TCX	MayoRNAseq									
		0.028	7.41E-01	FP	MSBB									
		0.117	**4.91E-02**	STG	MSBB									
		0.074	6.52E-02	DLPFC	ROSMAP									
		0.122	**7.50E-03**	PHG	MSBB									
		-0.135	**1.67E-02**	CBE	MayoRNAseq									
CERK	ENSG00000100422	0.058	1.84E-01	DLPFC	ROSMAP	0.160	0.065	**3.33E-02**	0.160	0.065	5.31E-02	5.580	0	1	0
		0.186	**2.15E-02**	CBE	MayoRNAseq									
		0.048	5.35E-01	IFG	MSBB									
		-0.044	5.20E-01	STG	MSBB									
		0.036	4.89E-01	PHG	MSBB									
		0.111	1.81E-01	TCX	MayoRNAseq									
		0.023	8.00E-01	FP	MSBB									
SPHK1	ENSG00000176170	0.08	7.58E-01	FP	MSBB	0.289	0.066	**8.59E-05**	0.295	0.089	**6.39E-03**	10.886	0.155	1.347	0.449
		0.208	3.27E-01	IFG	MSBB									
		0.689	**1.00E-04**	TCX	MayoRNAseq									
		0.113	3.39E-01	DLPFC	ROSMAP									
		0.379	**3.39E-02**	CBE	MayoRNAseq									
		0.49	**5.00E-04**	PHG	MSBB									

		0.004	9.87E-01	STG	MSBB										
CERS2	ENSG00000143418	0.043	8.29E-01	FP	MSBB	0.293	0.066	**6.75E-05**	0.293	0.066	**1.75E-04**	7.847	0.026	1.144	0.235
		0.131	1.59E-01	DLPFC	ROSMAP										
		0.241	**3.82E-02**	CBE	MayoRNAseq										
		0.107	4.45E-01	STG	MSBB										
		0.287	**1.12E-02**	TCX	MayoRNAseq										
		0.345	**5.00E-04**	PHG	MSBB										
		0.165	2.69E-01	IFG	MSBB										
KDSR	ENSG00000119537	0.058	4.70E-01	FP	MSBB	0.171	0.066	**2.33E-02**	0.193	0.141	3.20E-01	26.902	0.326	2.117	0.777
		0.167	**3.50E-03**	TCX	MayoRNAseq										
		-0.104	8.00E-02	CBE	MayoRNAseq										
		0.108	1.68E-01	IFG	MSBB										
		0.042	5.85E-01	STG	MSBB										
		0.039	3.50E-01	DLPFC	ROSMAP										
		0.15	**4.50E-03**	PHG	MSBB										
SPTLC3	ENSG00000172296	0.571	**4.60E-03**	TCX	MayoRNAseq	NA	NA	NA	NA	NA	NA	NA	NA	NA	NA
		0.443	**1.96E-02**	PHG	MSBB										
CERS1	ENSG00000223802	-0.091	3.27E-01	CBE	MayoRNAseq	0.327	0.066	**8.89E-06**	0.346	0.134	**3.98E-02**	23.858	0.305	1.994	0.749
		0.183	1.50E-01	STG	MSBB										
		0.062	7.26E-01	FP	MSBB										
		0.351	**4.00E-04**	PHG	MSBB										
		0.151	**1.74E-02**	DLPFC	ROSMAP										
		0.164	2.62E-01	IFG	MSBB										
		0.289	**5.00E-04**	TCX	MayoRNAseq										
SGMS1	ENSG00000198964	0.214	**1.20E-03**	TCX	MayoRNAseq	0.093	0.065	2.37E-01	0.082	0.112	6.36E-01	16.514	0.236	1.659	0.637
ACER3	ENSG00000078124	0.041	6.86E-01	IFG	MSBB	0.180	0.066	**1.61E-02**	0.167	0.101	2.20E-01	14.130	0.199	1.535	0.575
		0.088	1.47E-01	PHG	MSBB										
		0.029	8.00E-01	FP	MSBB										
		0.014	8.92E-01	STG	MSBB										
		-0.052	5.50E-01	CBE	MayoRNAseq										

		0.186	**1.41E-02**	TCX	MayoRNAseq										
		0.1	**2.62E-02**	DLPFC	ROSMAP										
DEGS1	ENSG00000143753	0.009	8.72E-01	CBE	MayoRNAseq	0.197	0.065	**8.25E-03**	0.197	0.065	**1.49E-02**	5.876	0.006	1	0
		0.059	6.73E-02	DLPFC	ROSMAP										
		0.071	8.52E-02	PHG	MSBB										
		0.022	7.60E-01	IFG	MSBB										
		0.053	2.79E-01	TCX	MayoRNAseq										
		0.045	4.58E-01	FP	MSBB										
		0.023	7.12E-01	STG	MSBB										
PLPP3	ENSG00000162407	0.174	2.51E-01	STG	MSBB	0.197	0.066	**8.38E-03**	0.218	0.118	1.63E-01	18.585	0.255	1.760	0.677
		-0.043	8.56E-01	FP	MSBB										
		-0.014	9.40E-01	CBE	MayoRNAseq										
		0.087	5.73E-01	DLPFC	ROSMAP										
		0.465	**1.00E-03**	TCX	MayoRNAseq										
		0.272	**1.75E-02**	PHG	MSBB										
		0.174	3.11E-01	IFG	MSBB										
SMPD3	ENSG00000103056	-0.233	**1.80E-03**	TCX	MayoRNAseq	-0248	0.066	**7.73E-04**	-0.272	0.107	**4.30E-02**	15.279	0.219	1.596	0.607
		-0.115	7.79E-02	PHG	MSBB										
		-0.102	2.36E-01	FP	MSBB										
		-0.081	3.59E-01	STG	MSBB										
		-0.038	5.46E-01	DLPFC	ROSMAP										
		-0.175	5.74E-02	IFG	MSBB										
		0.026	7.79E-01	CBE	MayoRNAseq										

**Pathway Enrichment Analysis**								
Total N = number of FDR-significant pathways for respective brain regions. EnrichR package v3.0 and KEGG database was used for enrichment analysis								
**Tissue**	**Total N**	**Term**	**Overlap**	**P-value**	**Adjusted P-value**	**Odds Ratio**	**Combined Score**	**Rank**
STG	49	Sphingolipid signaling pathway	12/119	0.00230194	0.028224279	2.80241891	17.0219055	19

TCX	71	Sphingolipid signaling pathway	57/119	0.00262653	0.019617711	1.70675056	10.1416678	42
PHG	75	Sphingolipid signaling pathway	47/119	0.0024407	0.015839638	1.74919798	10.5222494	49
CBE	0							
FP	0							
IFG	0							
DLPFC	0							

**DEG ranking list**							
Rank of each gene based on significance of differential expression in AD vs controls. Only FDR-significant genes were included in this analysis, and the total number is shown in the last row.							
**Gene ID**	**CBE**	**TCX**	**FP**	**STG**	**PHG**	**IFG**	**DLPFC**
ENSG00000090054	1854	3409					
ENSG00000100596							
ENSG00000172296		3996			3980		
ENSG00000119537		3736			2394		
ENSG00000223802		2429			796		974
ENSG00000143418	5619	4934			993		
ENSG00000154227							
ENSG00000090661	3051	2540	243		5313		1321
ENSG00000139624	3907						
ENSG00000172292	351				3981		
ENSG00000143753							
ENSG00000168350							
ENSG00000167769							
ENSG00000177076	1609	6792					
ENSG00000078124		5200					1233
ENSG00000104763							
ENSG00000188611							
ENSG00000198964		2937					

ENSG00000164023							
ENSG00000166311							
ENSG00000135587							
ENSG00000103056		3206					
ENSG00000136699	3259						462
ENSG00000182156							
ENSG00000100422	4807						
ENSG00000067113	3454	866			878		572
ENSG00000162407		2769			3807		
ENSG00000141934							
ENSG00000148154							
ENSG00000177628							
ENSG00000176170	5421	1426			991		
ENSG00000063176							
ENSG00000126821							
ENSG00000163082	1933						
ENSG00000166224	4536	2501		730	2882		
Total N significant genes	6059	7017	364	779	5450	169	1860

Abbreviations: DLPFC: Dorsolateral Prefrontal Cortex; CBE: Cerebellum; DEG: Differentially expressed genes; FDR: False discovery rate; FP: Frontal pole; IFG: Interior Frontal Gyrus; MayoRNAseq: Mayo clinic RNA-seq study; MSBB: Mount Sinai Brain Bank; PHG: Parahippocampal Gyrus; ROSMAP: Religious Orders Study/Memory and Aging Project; SM: Sphingomyelin; STG: Superior Temporal Gyrus; TCX: Temporal Cortex.

Supplementary Table 3. Genetic variants in the coding regions of genes in SM pathway associated with AD and A-T-N-C measures. Gene-based association analysis of ADNI individuals were used for identifying the genetic variants.

Gene Symbol	Phenotype	Lead SNP	Position (Grch37)	Effect Allele	Effect Direction	p-value (Not Adjusted for APOE E4)	p-value (Adjusted for APOE E4)	p-value Threshold	Link to Gene	Eqtl Direction	Eqtl Source
ACER2	CSF p-tau/Aβ_{1-42}	rs10118158	chr9: 19,411,179	T	+	0.0006625	1.10E-05	2.82E-05	location (intron)		
	CSF t-tau/Aβ_{1-42}	rs10118158	chr9: 19,411,179	T	+	0.0006048	9.29E-06	2.82E-05	location (intron)		
CERS2	CSF-Abeta	rs79427717	chr1: 150,203,716	A	-	4.80E-05	8.20E-05	0.0001073	eQTL	-	GTEx v8
CERS3	FDG-PET (ROI-based)	rs1847325	chr15: 101,067,542	C	+	8.78E-06	1.49E-05	9.85E-06	eQTL; location (intron)	-	GTEx v8
	No AD vs. AD	rs8035180	chr15: 100,995,603	C	+	7.18E-06	-	9.85E-06	eQTL; location (intron)	+	GTEx v8
CERS6	ADAS-Cog13	rs829963	chr2: 169,601,513	G	-	6.40E-09	1.35E-07	5.43E-06	eQTL; location (intron)	+	GTEx v8
	ADNI-Executive-Function	rs829963	chr2: 169,601,513	G	+	8.69E-07	9.89E-06	5.43E-06	eQTL; location (intron)	+	GTEx v8
	ADNI-Memory Score	rs829963	chr2: 169,601,513	G	+	2.74E-08	6.25E-07	5.43E-06	eQTL; location (intron)	+	GTEx v8
	RAVLT total score	rs829963	chr2: 169,601,513	G	+	3.32E-06	2.81E-05	5.43E-06	eQTL; location (intron)	+	GTEx v8
	CN vs. AD	rs829963	chr2: 169,601,513	G	-	5.26E-07	-	5.43E-06	eQTL; location (intron)	+	GTEx v8

	No AD vs. AD	rs145495456	chr2: 169,602,046	C	-	2.24E-08	-	5.43E-06	location (intron)		
DEGS1	ADAS-Cog13	rs12737029	chr1: 224,316,205	C	-	3.66E-05	5.03E-05	5.39E-05	eQTL	-	GTEx v8
	CN vs. AD	rs12737029	chr1: 224,316,205	C	-	5.44E-07	-	5.39E-05	eQTL	-	GTEx v8
	No AD vs. AD	rs12737029	chr1: 224,316,205	C	-	1.93E-05	-	5.39E-05	eQTL	-	GTEx v8
PLPP2	CSF p-tau/Aβ_{1-42}	rs12982096	chr19: 288,738	T	-	6.84E-06	0.006781	6.11E-05	eQTL; location (intron)	+	GTEx v8
	CSF t-tau/Aβ_{1-42}	rs12982096	chr19: 288,738	T	-	8.15E-06	0.007858	6.11E-05	eQTL; location (intron)	+	GTEx v8
SPHK2	FDG-PET (ROI-based)	rs281380	chr19: 49,214,470	C	+	4.15E-05	8.58E-05	7.35E-05	eQTL	-	GTEx v8
SMPD2	clinico-pathologic AD dementia*	rs3799845	chr6: 110,027,717	A	+	3.53E-05	-	3.78E-05	eQTL	+	GTEx v8
SGMS1	FDG-PET (ROI-based)**	rs372273418	chr10: 52,382,799	A	+	5.35E-05	0.000196	5.83E-06	location (intron)		
SPTLC3	RAVLT total score**	rs2327754	chr20: 13,103,159	T	-	1.00E-04	6.71E-05	9.77E-06	location (intron)		

* External (Beecham et al.)
** Significant only in permutation test
Abbreviations: AD: Alzheimer's Disease; ADAS-Cog.13: Alzheimer's Disease Assessment Scale - Cognitive Subscale; ADNI: Alzheimer's Disease Neuroimaging Initiative; A-T-N-C: Apparent Total N-nitroso Compounds; chr: Chromosome; CN: Cognitively Normal; CSF: Cerebrospinal Fluid; eQTL: expression Quantitative Trait Loci; FDG: Fluorodeoxyglucose; GTEx: Genotype-Tissue Expression project; PET: Positron Emission Tomography; RAVLT: Rey Auditory Verbal Learning Test; SM: Sphingomyelin; SNP: Single nucleotide polymorphism.

Supplementary Table 4. Genome-wide and gene-wide significant associations for 14 related SMs for six genes in the pathway.

Gene Symbol	Metabolite	Lead SNP	Position (Grch37)	Effect Allele	Effect Direction	Min. p-value	Source	p-value Threshold	link to gene	eQTL direction	eQTL source
CERS2	sphingomyelin (d18:1/15:0, d16:1/17:0)	rs7548516	chr1: 150,980,281	T	-	4.60E-05	Shin et al.	1.07E-04	eQTL	-	GTEx v8
	SM C16:0	rs11204752	chr1: 150,958,133	T	-	1.22E-05	ADNI-1/GO/2	1.07E-04	eQTL	-	GTEx v8
	SM C16:1	rs1673160	chr1: 150,970,836	T	+	4.45E-05	ADNI-1/GO/2	1.07E-04	eQTL	+	Westra et al.
	SM C24:0	rs11204752	chr1: 150,958,133	T	-	5.28E-05	ADNI-1/GO/2	1.07E-04	eQTL	-	GTEx v8
	SM C26:0	rs11204752	chr1: 150,958,133	T	-	2.01E-06	ADNI-1/GO/2	1.07E-04	eQTL	-	GTEx v8
	SM (OH) C22:1	rs771201	chr1: 150,968,942	C	+	8.95E-06	ADNI-1/GO/2	1.07E-04	eQTL	+	GTEx v6
	SM (OH) C24:1	rs11204752	chr1: 150,958,133	T	-	7.48E-06	ADNI-1/GO/2	1.07E-04	eQTL	-	GTEx v8
CERS4	SM C18:0	rs12610250	chr19: 8,277,713	A	-	9.87E-26	Draisma et al.	2.33E-05	eQTL; location (intron)	-	GTEx v8
	SM C18:1	rs12610250	chr19: 8,277,713	A	-	1.28E-21	Draisma et al.	2.33E-05	eQTL; location (intron)	-	GTEx v8
	SM C20:2	rs10418768	chr19: 8,280,621	C	-	6.94E-09	Draisma et al.	2.33E-05	eQTL; location (intron)	-	GTEx v8
SGPL1	SM C16:1	rs11594636	chr10: 72,610,767	A	+	1.16E-05	Draisma et al.	1.94E-05	eQTL; location (intron)	-	GTEx v8
	SM C18:1	rs2630339	chr10: 72,574,353	C	+	3.41E-06	Draisma et al.	1.94E-05	eQTL	-	GTEx v8
SGPP1	sphingomyelin (d18:1/14:0, d16:1/16:0)	rs12891564	chr14: 64,435,939	A	+	5.60E-31	Shin et al.	3.55E-05	eQTL	+	GTEx v8
	sphingomyelin (d18:1/15:0, d16:1/17:0)	rs12891564	chr14: 64,435,939	A	+	7.93E-17	Shin et al.	3.55E-05	eQTL	+	GTEx v8
	SM (OH) C14:1	rs12589453	chr14: 64,376,864	A	+	3.55E-23	Draisma et al.	3.55E-05	eQTL	+	GTEx v8
SPHK2	sphingomyelin (d18:1/14:0, d16:1/16:0)	rs601338	chr19: 49,206,674	A	+	2.01E-05	Shin et al.	7.35E-05	eQTL	-	GTEx v8

SPTLC3	sphingomyelin (d18:1/14:0, d16:1/16:0)	rs4814176	chr20: 12,959,398	T	+	2.97E-17	Shin et al.	9.77E-06	eQTL	+	GTEx v8
	sphingomyelin (d18:1/15:0, d16:1/17:0)	rs4814176	chr20: 12,959,398	T	+	1.04E-09	Shin et al.	9.77E-06	eQTL	+	GTEx v8
	SM C26:0	rs680379	chr20: 12,969,400	A	+	8.49E-15	Draisma et al.	9.77E-06	eQTL	+	GTEx v8
	SM C26:1	rs4814176	chr20: 12,959,398	T	+	3.34E-13	Draisma et al.	9.77E-06	eQTL	+	GTEx v8
	SM (OH) C14:1	rs4814176	chr20: 12,959,398	T	+	2.28E-11	Draisma et al.	9.77E-06	eQTL	+	GTEx v8
	SM (OH) C16:1	rs4814176	chr20: 12,959,398	T	+	3.95E-13	Draisma et al.	9.77E-06	eQTL	+	GTEx v8
	SM (OH) C24:1	rs4814176	chr20: 12,959,398	T	+	2.01E-32	Draisma et al.	9.77E-06	eQTL	+	GTEx v8
*SPTLC3**	SM (OH) C24:1/SM C16:0	rs680379	chr20: 12,969,400	A	+	1.01E-09	ADNI-1	9.77E-06	eQTL	+	GTEx v8
		rs168622	chr20: 12,966,089	T	+	5.20E-25	Illig et al.	9.77E-06	eQTL	+	GTEx v8

* Ratio analysis

Abbreviations: ADNI: Alzheimer's Disease Neuroimaging; chr: Chromosome; eQTL: expression Quantitative Trait Loci; GTEx: Genotype-Tissue Expression project; SM: Sphingomyelin species; SNP: Single Nucleotide Polymorphism.

Supplementary Table 5. Statistical analysis of Figure 5 and Supplementary Figure 2.

Phenotype	n	Selected Covariates	Metabolite*	Estimate	Std. Error	Statistic	p-value	p-gain
Clinical AD	373	Age, sex, education, copies of APOE4	SM C16:0	1.0075	0.0034	-2.1654	3.04E-02	
			SM (OH) C24:1	0.8494	0.2235	0.7304	4.65E-01	
			**ratio**	**1.9453**	**0.5174**	**3.7596**	**1.70E-04**	**178.37**
ADAS-Cog. 13	725	Age, sex, education	SM C16:0	0.0243	0.0095	2.5460	1.11E-02	
			SM (OH) C24:1	-0.4983	0.6301	-0.7908	4.29E-01	
			**ratio**	**-6.4817**	**1.4003**	**-4.6288**	**4.36E-06**	**2544.51**
SPARE-AD	731	Age, sex	SM C16:0	0.0037	0.0013	2.7996	5.25E-03	
			SM (OH) C24:1	-0.0649	0.0876	-0.7407	4.59E-01	
			**ratio**	**-0.8879**	**0.1970**	**-4.5077**	**7.64E-06**	**687.57**

* Results for single metabolites are from Toledo et al.[1]

Abbreviations: AD: Alzeimer's Disease; ADAS-Cog.13: Alzheimer's Disease Assessment Scale - Cognitive Subscale; SM: Sphingomyelin species; SPARE-AD: Spatial Pattern of Abnormality for Recognition of Early Alzheimer's Disease.

Supplementary Table 6. GWAS analyses for A-T-N-C measures, clinical diagnosis and metabolites levels in 1,576 individuals.

Experiment	Comparison	Statistical Test	Adjusted p-value
	Novel Object Recognition: Discrimination index. **Figure 5A.**	One-way ANOVA $F_{(3,51)} = 5.715$	0.002
**FTY720 ameliorates memory impairment in APP/PS1 mice.**	WT Vehicle vs. APP/PS1 Vehicle	Tukey's post hoc. $q_{(51)} = 4.528$	0.0122
**Figure 5.**	WT Vehicle vs. WT + FTY720	Tukey's post hoc. $q_{(51)} = 0.1099$	0.9998
	WT Vehicle vs. APP/PS1 + FTY720	Tukey's post hoc. $q_{(51)} = 0.3512$	0.9946
	APP/PS1 Vehicle vs. WT + FTY720	Tukey's post hoc. $q_{(51)} = 4.751$	0.0078
	APP/PS1 Vehicle vs. APP/PS1 + FTY720	Tukey's post hoc. $q_{(51)} = 3.993$	0.0332
	WT + FTY720 vs. APP/PS1 + FTY720	Tukey's post hoc. $q_{(51)} = 0.2637$	0.9977
	Novel Object Recognition Discrimination index against chance (Zero). **Figure 5A.**	One sample t-test	
**FTY720 ameliorates memory impairment in APP/PS1 mice. Figure 5.**	WT Vehicle vs. Zero	$t_{(10)} = 4.802$	0.0007
	WT + FTY720 vs. Zero	$t_{(13)} = 3.546$	0.0036
	APP/PS1 Vehicle vs. Zero	$t_{(19)} = 0.2171$	0.8304
	APP/PS1 + FTY720 vs. Zero	$t_{(9)} = 11.54$	<0.0001
	Barnes Maze Latency to find Escape hole. **Figure 5B.**	Two-way Repeated Measure ANOVA $F_{(9,201)} = 1.223$	0.2827
	**Day 1**		
	WT Vehicle vs. WT+FTY720	Tukey's post hoc. $q_{(201)} = 1.807$	0.5846
	WT Vehicle vs. APP/PS1 Vehicle	Tukey's post hoc. $q_{(201)} = 2.507$	0.3177
	WT Vehicle vs. APP/PS1 + FTY720	Tukey's post hoc. $q_{(201)} = 0.4919$	0.9851

	WT+FTY720 vs. APP/PS1 Vehicle	Tukey's post hoc. $q_{(201)} = 0.3258$	0.9956
	WT+FTY720 vs. APP/PS1 + FTY720	Tukey's post hoc. $q_{(201)} = 1.794$	0.5904
	APP/PS1 Vehicle vs. APP/PS1 + FTY720	Tukey's post hoc. $q_{(201)} = 3.110$	0.1415
	**Day 2**		
	WT Vehicle vs. WT+FTY720	Tukey's post hoc. $q_{(201)} = 0.8049$	0.9404
**FTY720 ameliorates memory impairment in APP/PS1 mice.**	WT Vehicle vs. APP/PS1 Vehicle	Tukey's post hoc. $q_{(201)} = 2.780$	0.2319
**Figure 5.**	WT Vehicle vs. APP/PS1 + FTY720	Tukey's post hoc. $q_{(201)} = 1.724$	0.6196
	WT+FTY720 vs. APP/PS1 Vehicle	Tukey's post hoc. $q_{(201)} = 1.696$	0.6326
	WT+FTY720 vs. APP/PS1 + FTY720	Tukey's post hoc. $q_{(201)} = 0.9309$	0.9119
	APP/PS1 Vehicle vs. APP/PS1 + FTY720	Tukey's post hoc. $q_{(201)} = 0.4718$	0.9869
	**Day 3**		
	WT Vehicle vs. WT+FTY720	Tukey's post hoc. $q_{(201)} = 1.998$	0.5045
	WT Vehicle vs. APP/PS1 Vehicle	Tukey's post hoc. $q_{(201)} = 6.667$	0.0003
	WT Vehicle vs. APP/PS1 + FTY720	Tukey's post hoc. $q_{(201)} = 3.988$	0.0394
	WT+FTY720 vs. APP/PS1 Vehicle	Tukey's post hoc. $q_{(201)} = 5.741$	0.0019
	WT+FTY720 vs. APP/PS1 + FTY720	Tukey's post hoc. $q_{(201)} = 2.757$	0.2305
	APP/PS1 Vehicle vs. APP/PS1 + FTY720	Tukey's post hoc. $q_{(201)} = 2.688$	0.2447
	**Day 4**		
	WT Vehicle vs. WT+FTY720	Tukey's post hoc. $q_{(201)} = 1.243$	0.8157
	WT Vehicle vs. APP/PS1 Vehicle	Tukey's post hoc. $q_{(201)} = 5.114$	0.0056
	WT Vehicle vs. APP/PS1 + FTY720	Tukey's post hoc. $q_{(201)} = 1.945$	0.5242

	WT+FTY720 vs. APP/PS1 Vehicle	Tukey's post hoc. $q_{(201)} = 3.702$	0.0598
	WT+FTY720 vs. APP/PS1 + FTY720	Tukey's post hoc. $q_{(201)} = 1.002$	0.8931
	APP/PS1 Vehicle vs. APP/PS1 + FTY720	Tukey's post hoc. $q_{(201)} = 1.754$	0.6066
	Barnes Maze<br>Time spent in Escape hole.<br>**Figure 5C.**	Kruskal-Wallis test<br>Chi square = 19.93	0.0002
**FTY720 ameliorates memory impairment in APP/PS1 mice.**	WT Vehicle vs. APP/PS1 Vehicle	Dunn's post hoc<br>Z = 3.698	0.0013
**Figure 5.**	WT Vehicle vs. WT + FTY720	Dunn's post hoc<br>Z = 0.5109	>0.9999
	WT Vehicle vs. APP/PS1 + FTY720	Dunn's post hoc<br>Z = 0.6318	>0.9999
	APP/PS1 Vehicle vs. WT + FTY720	Dunn's post hoc<br>Z = 3.424	0.0037
	APP/PS1 Vehicle vs. APP/PS1 + FTY720	Dunn's post hoc<br>Z = 3.404	0.004
	WT + FTY720 vs. APP/PS1 + FTY720	Dunn's post hoc<br>Z = 0.1174	>0.9999
	Barnes Maze<br>Time spent in Escape hole against chance (0.05).<br>**Figure 5C.**	One sample t-test	
**FTY720 ameliorates memory impairment in APP/PS1 mice.<br>Figure 5.**	WT Vehicle vs. 0.05	$t_{(12)} = 8.396$	<0.0001
	WT + FTY720 vs. 0.05	$t_{(16)} = 5.587$	<0.0001
	APP/PS1 Vehicle vs. 0.05	$t_{(20)} = 6.559$	<0.0001
	APP/PS1 + FTY720 vs. 0.05	$t_{(18)} = 7.461$	<0.0001
	CA3-CA1 synapse LTP<br>Magnitude of LTP between 60 and 75 min after the HFS.<br>**Figure 5E.**	Two-way ANOVA<br>$F_{(1.36)} = 8.975$	0.0049
	Vehicle:WT vs. Vehicle:APP/PS1	Tukey's post hoc. $q_{(36)} = 4.575$	0.0133
**FTY720 ameliorates synaptic impairment in APP/PS1 mice.**	Vehicle:WT vs. FYT720:WT	Tukey's post hoc. $q_{(36)} = 0.2969$	0.9967

**Figure 5.**			
	Vehicle:WT vs. FYT720:APP/PS1	Tukey's post hoc. $q_{(36)} = 1.285$	0.8005
	Vehicle:APP/PS1 vs. FYT720:WT	Tukey's post hoc. $q_{(36)} = 4.001$	0.0364
	Vehicle:APP/PS1 vs. FYT720:APP/PS1	Tukey's post hoc. $q_{(36)} = 5.587$	0.0019
	FYT720:WT vs. FYT720:APP/PS1	Tukey's post hoc. $q_{(36)} = 1.482$	0.7227
	LECII-LECII synapse LTP Magnitude of LTP between 60 and 75 min after the HFS. **Figure 5G.**	Two-way ANOVA $F_{(1,24)} = 12.34$	0.0018
	Vehicle:WT vs. Vehicle:APP/PS1	Tukey's post hoc. $q_{(24)} = 7.440$	0.0001
**FTY720 ameliorates synaptic impairment in APP/PS1 mice. Figure 5.**	Vehicle:WT vs. FYT720:WT	Tukey's post hoc. $q_{(24)} = 2.208$	0.4187
	Vehicle:WT vs. FYT720:APP/PS1	Tukey's post hoc. $q_{(24)} = 2.623$	0.2735
	Vehicle:APP/PS1 vs. FYT720:WT	Tukey's post hoc. $q_{(24)} = 5.232$	0.0058
	Vehicle:APP/PS1 vs. FYT720:APP/PS1	Tukey's post hoc. $q_{(24)} = 4.816$	0.0116
	FYT720:WT vs. FYT720:APP/PS1	Tukey's post hoc. $q_{(24)} = 0.4155$	0.9909
**FTY720 ameliorates memory impairment in APP/PS1 mice. Supplementary Figure 2.**	Novel Object Recognition Discrimination index. **Supplementary Figure 2A.**	Unpaired two-sided Student's t-test	
	APP/PS1 7mo vs. WT 7mo	$t_{(14)} = 2.230$	0.0426
**FTY720 ameliorates memory impairment in APP/PS1 mice. Supplementary Figure 2.**	Novel Object Recognition Discrimination index against chance (Zero). **Supplementary Figure 2A.**	One sample t-test	
	WT 7mo vs. Zero	$t_{(4)} = 4.407$	0.0116
	APP/PS1 7mo vs. Zero	$t_{(10)} = 0.3131$	0.7606

	Barnes Maze<br>Latency to find Escape hole.<br>**Supplementary Figure 2B.**	Two-way Repeated Measure ANOVA<br>$F_{(3.36)}$ = 0.7277	0.5422
**FTY720 ameliorates memory impairment in APP/PS1 mice.**	**Day 1**		
**Supplementary Figure 2.**	WT 7mo - APP/PS1 7mo	Šídák's post hoc<br>$t_{(36)}$ = 3.098	0.015
	**Day 2**		
	WT 7mo - APP/PS1 7mo	Šídák's post hoc<br>$t_{(36)}$ = 3.156	0.0128
	**Day 3**		
	WT 7mo - APP/PS1 7mo	Šídák's post hoc<br>$t_{(36)}$ = 1.999	0.1964
	**Day 4**		
	WT 7mo - APP/PS1 7mo	Šídák's post hoc<br>$t_{(36)}$ = 1.418	0.5137
**FTY720 ameliorates memory impairment Supplementary Figure 2.**	Barnes Maze<br>Time spent in Escape hole.<br>**Supplementary Figure 2C.**	Unpaired two-sided Student's t-test	
	APP/PS1 7mo Vs. WT 7mo	$t_{(7)}$ = 3.131	0.0166
**FTY720 ameliorates memory impairment in APP/PS1 mice. Supplementary Figure 2.**	Barnes Maze<br>Time spent in Escape hole against chance (0.05).<br>**Supplementary Figure 2C.**	One sample t-test	
	WT 7mo vs. Zero	$t_{(3)}$ = 5.695	0.0107
	APP/PS1 7mo vs. Zero	$t_{(4)}$ = 4.264	0.013
**FTY720 ameliorates synaptic impairment in APP/PS1 mice. Supplementary Figure 2.**	CA3-CA1 synapse LTP<br>Magnitude of LTP between 60 and 75 min after the HFS.<br>**Supplementary Figure 2E.**<br>APP/PS1 7mo vs. WT 7mo	Unpaired two-sided Student's t-test<br>$t_{(13)}$ = 5.197	0.0002
**FTY720 ameliorates synaptic impairment in APP/PS1 mice. Supplementary Figure 2.**	LECII-LECII synapse LTP<br>Magnitude of LTP between 60 and 75 min after the HFS<br>**Supplementary Figure 2G.**	Unpaired two-sided Student's t-test	
	APP/PS1 7mo Vs. WT 7mo	$t_{(6)}$ = 4.918	0.0027

Abbreviations: ANOVA: Analysis of Variance; A-T-N-C: Apparent Total N-nitroso Compounds; GWAS: Genome-Wide Association Study; LTP: Long-Term Potentiation; WT: Wild Type.

Supplementary Figure 1. Association of genetic variants in *CERS3* with CSF t-tau biomarker.

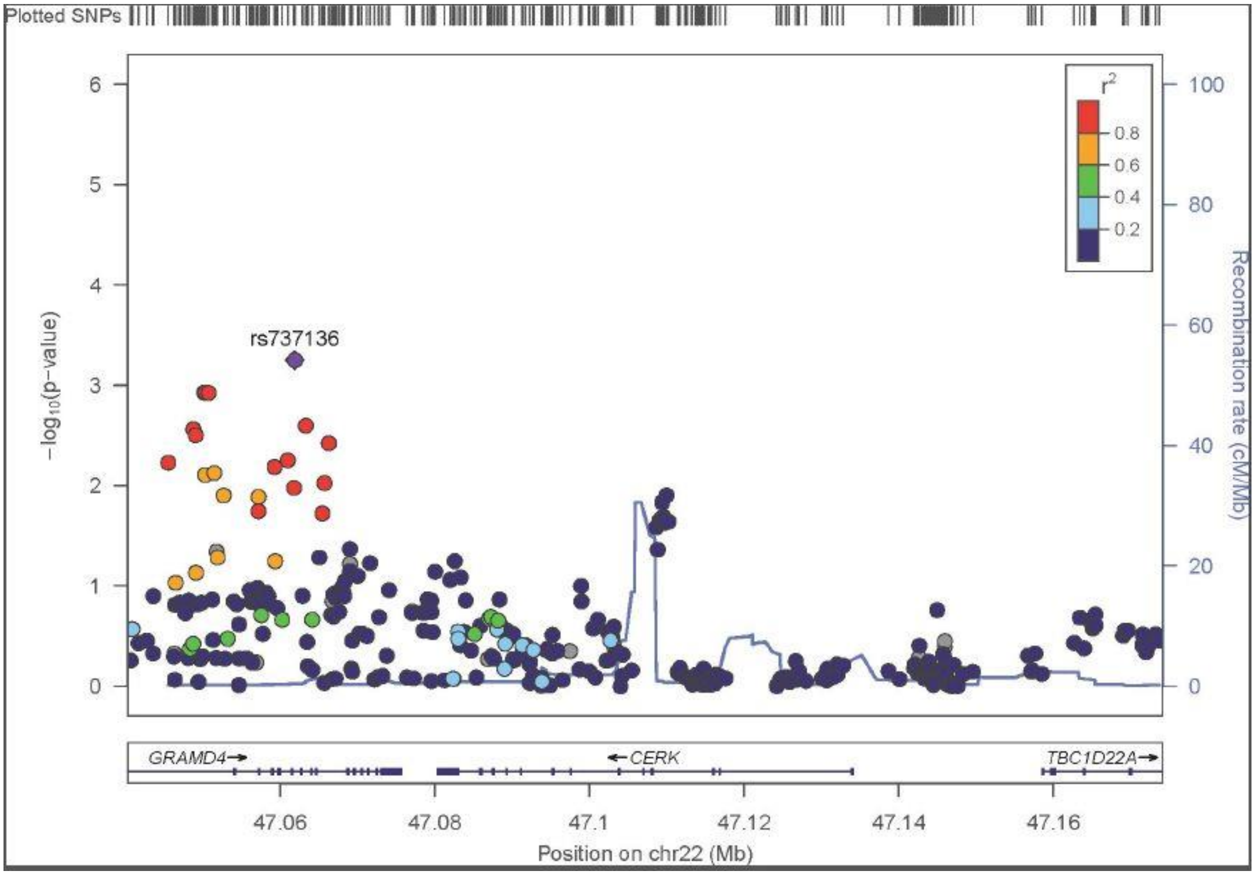

Abbreviation: chr: Chromosome.

Supplementary Figure 2. Comparative analysis of memory and synaptic plasticity in APP/PS1 and wildtype 7 months old mice.

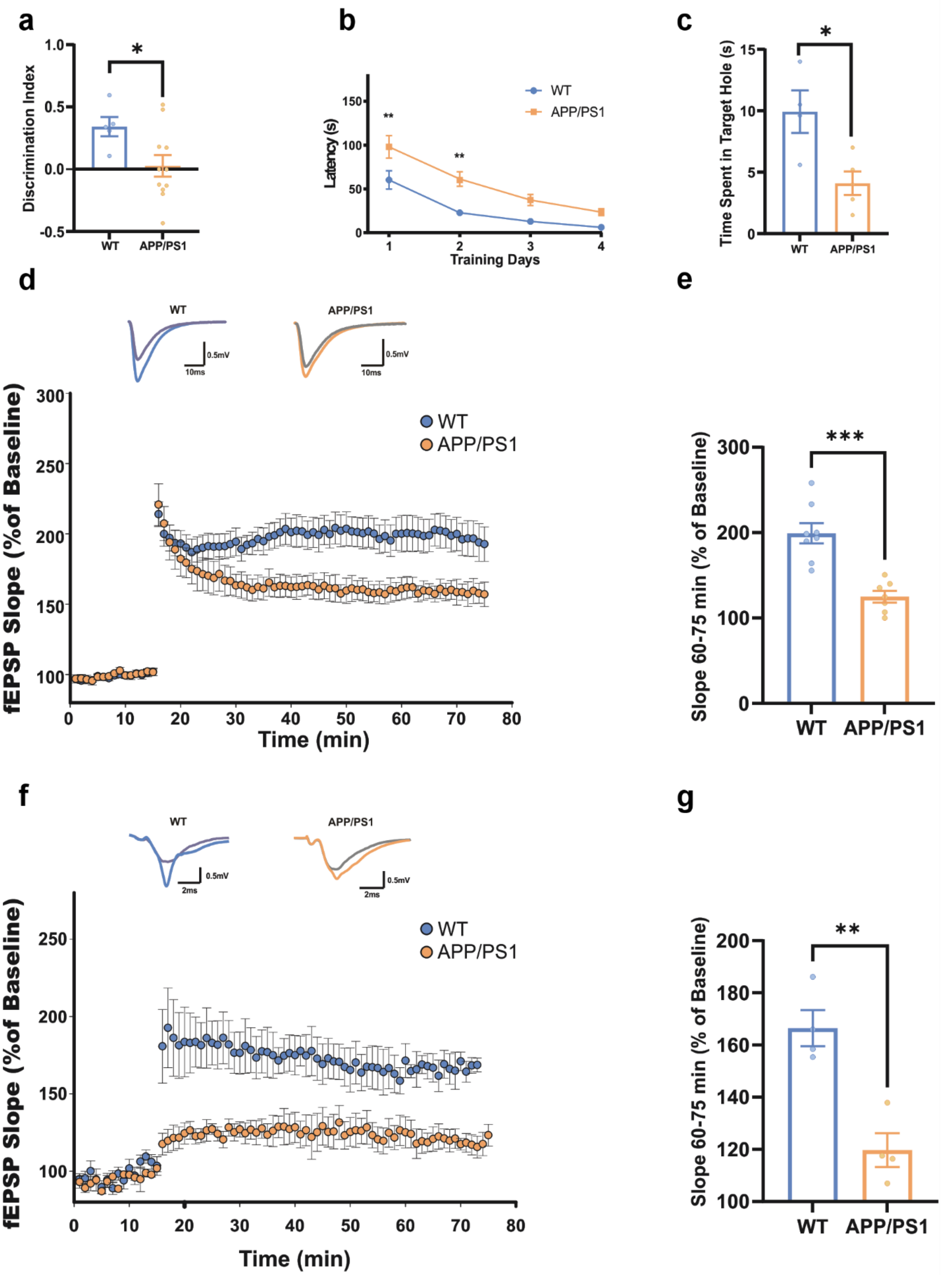

a. Exploration time spent on the novel object in the NOR test session. Data are expressed as a discrimination index ± SEM. **b**. Barnes maze performance during training days. The average time (in seconds) to locate the escape hole during the four trials per day was expressed in mean ± SEM. **c.** The time spent in the target hole is plotted ± SEM. **d.** LTP assessment at the CA3-CA1 synapse. The two small line graphs are representative analog traces of evoked EPSPs before (light purple and grey) and 1 hr. after high-frequency stimulation (HFS) (blue and orange). The large plot graph shows normalized evoked excitatory postsynaptic potentials (EPSPs) slopes in APP/PS1 and WT mice before (first 15 min. of baseline) and after HFS (HFS, delivered at 16-min). **e.** Magnitude of LTP between 60 and 75 min after HFS. Data represent mean fEPSP Slope ± SEM. APP/PS1 mice showed reduced LTP in the CA3-CA1 synapse. **f**. LTP assessment at the LECII-LECII synapse. The two small line graphs are representative analog traces of evoked EPSPs before (light purple and grey) and 1 hr. after HFS (blue and orange). The large plot graph is an LTP timeline. **g.** Magnitude of LTP between 60 and 75 min after HFS. Data represent mean fEPSP Slope ± SEM. APP/PS1 mice showed reduced LTP in the LECII-LECII synapse. $*p < 0.05$, $**p < 0.01$, and $***p < 0.001$. *Abbreviations:* fEPSP: Field excitatory post-synaptic potentials; WT: Wild type.

Supplementary References

1. Toledo, J. B. *et al.* Metabolic network failures in Alzheimer's disease: A biochemical road map. *Alzheimers. Dement.* **13**, 965–984 (2017).
