# Supplementary material for: Multi-Omic analyses characterize the ceramide/sphingomyelin pathway as a therapeutic target in Alzheimer’s disease
Source: Commun Biol. 2022 Oct 8;5:1074. doi: 10.1038/s42003-022-04011-6 (PMC9547905; doi:10.1038/s42003-022-04011-6)
Supplement: Supplementary file 2 — Description of Additional Supplementary Files [file 42003_2022_4011_MOESM2_ESM.pdf]

Description of Additional Supplementary Files

File name: Supplementary Data 1
Description: A listing of the sphingolipids profiled in ADNI1 individuals.
