# Supplementary material for: Multi-Omic analyses characterize the ceramide/sphingomyelin pathway as a therapeutic target in Alzheimer’s disease
Source: Commun Biol. 2022 Oct 8;5:1074. doi: 10.1038/s42003-022-04011-6 (PMC9547905; doi:10.1038/s42003-022-04011-6)
Supplement: Supplementary file 4 — Reporting Summary [file 42003_2022_4011_MOESM4_ESM.pdf]

nature portfolio

Corresponding author(s): Xianlin Han and Rima Kaddurah-Daouk

Last updated by author(s): Sep 8, 2022

Reporting Summary

Nature Portfolio wishes to improve the reproducibility of the work that we publish. This form provides structure for consistency and transparency in reporting. For further information on Nature Portfolio policies, see our Editorial Policies and the Editorial Policy Checklist.

Statistics

For all statistical analyses, confirm that the following items are present in the figure legend, table legend, main text, or Methods section.

n/a	Confirmed	
☐	☒	The exact sample size (n) for each experimental group/condition, given as a discrete number and unit of measurement
☐	☒	A statement on whether measurements were taken from distinct samples or whether the same sample was measured repeatedly
☐	☒	The statistical test(s) used AND whether they are one- or two-sided<br>*Only common tests should be described solely by name; describe more complex techniques in the Methods section.*
☐	☒	A description of all covariates tested
☐	☒	A description of any assumptions or corrections, such as tests of normality and adjustment for multiple comparisons
☐	☒	A full description of the statistical parameters including central tendency (e.g. means) or other basic estimates (e.g. regression coefficient) AND variation (e.g. standard deviation) or associated estimates of uncertainty (e.g. confidence intervals)
☐	☒	For null hypothesis testing, the test statistic (e.g. F, t, r) with confidence intervals, effect sizes, degrees of freedom and P value noted<br>*Give P values as exact values whenever suitable.*
☒	☐	For Bayesian analysis, information on the choice of priors and Markov chain Monte Carlo settings
☒	☐	For hierarchical and complex designs, identification of the appropriate level for tests and full reporting of outcomes
☐	☒	Estimates of effect sizes (e.g. Cohen's d, Pearson's r), indicating how they were calculated

Our web collection on statistics for biologists contains articles on many of the points above.

Software and code

Policy information about availability of computer code

Data collection	The data was collected with permission from AMP-AD Knowledge Portal.
Data analysis	We used COBRA toolbox v3.069 for metabolic analysis that was implemented in MATLAB R2018a and academic licenses of Gurobi optimizer v7.5 and IBM CPLEX v12.7.1 were used to solve LP and MILP problems. For surface-based whole brain analysis of cortical thickness on a vertex-by-vertex basis, the SurfStat software package (www.math.mcgill.ca/keith/surfstat/) was used to perform a multivariable analysis. Genetic associations were calculated using PLINK v1.9. For mouse experiments, data was analyzed statistically using repeated measures ANOVA with the SPSS package.

For manuscripts utilizing custom algorithms or software that are central to the research but not yet described in published literature, software must be made available to editors and reviewers. We strongly encourage code deposition in a community repository (e.g. GitHub). See the Nature Portfolio guidelines for submitting code & software for further information.

Data

Policy information about availability of data

All manuscripts must include a data availability statement. This statement should provide the following information, where applicable:

- Accession codes, unique identifiers, or web links for publicly available datasets
- A description of any restrictions on data availability
- For clinical datasets or third party data, please ensure that the statement adheres to our policy

Metabolomics datasets from the AbsoluteIDQ-p180 metabolomics kit used in the current analyses for the ADNI-1 and ADNI-GO/-2 cohorts as well as the RNASeq data from the ROS/MAP, Mount Sinai Brain Bank Cohort, and the Mayo Clinic cohort are available via the Accelerating Medicines Partnership-Alzheimer's Disease (AMP-AD) Knowledge Portal and can be accessed at http://dx.doi.org/10.7303/syn5592519 (ADNI-1), http://dx.doi.org/10.7303/syn9705278 (ADNI-GO/-2), https://doi.org/10.7303/syn3388564 (ROS/MAP), https://doi.org/10.7303/syn3157743 (MSSB), and https://doi.org/10.7303/syn5550404 (Mayo clinic). The full complement of clinical and demographic data for the ADNI cohorts are hosted on the LONI data sharing platform and can be requested at http://adni.loni.usc.edu/data-samples/access786 data/. The full complement of clinical and demographic data for the ROS/MAP cohorts are available via the Rush AD Center Resource Sharing Hub and can be requested at https://www.radc.rush.edu. No customized codes were used for analyses.

Human research participants

Policy information about studies involving human research participants and Sex and Gender in Research.

Reporting on sex and gender	*Use the terms sex (biological attribute) and gender (shaped by social and cultural circumstances) carefully in order to avoid confusing both terms. Indicate if findings apply to only one sex or gender; describe whether sex and gender were considered in study design whether sex and/or gender was determined based on self-reporting or assigned and methods used. Provide in the source data disaggregated sex and gender data where this information has been collected, and consent has been obtained for sharing of individual-level data; provide overall numbers in this Reporting Summary. Please state if this information has not been collected. Report sex- and gender-based analyses where performed, justify reasons for lack of sex- and gender-based analysis.*
Population characteristics	*Describe the covariate-relevant population characteristics of the human research participants (e.g. age, genotypic information, past and current diagnosis and treatment categories). If you filled out the behavioural & social sciences study design questions and have nothing to add here, write "See above."*
Recruitment	*Describe how participants were recruited. Outline any potential self-selection bias or other biases that may be present and how these are likely to impact results.*
Ethics oversight	*Identify the organization(s) that approved the study protocol.*

Note that full information on the approval of the study protocol must also be provided in the manuscript.

Field-specific reporting

Please select the one below that is the best fit for your research. If you are not sure, read the appropriate sections before making your selection.

☒ Life sciences ☐ Behavioural & social sciences ☐ Ecological, evolutionary & environmental sciences

For a reference copy of the document with all sections, see nature.com/documents/nr-reporting-summary-flat.pdf

Life sciences study design

All studies must disclose on these points even when the disclosure is negative.

Sample size	265 post-mortem brain samples of temporal cortex (TC) and cerebellum (CER), 632 samples of frontal cortex (FC), 303 samples of frontal pole (FP), superior temporal gyrus (STG), inferior frontal gyrus (IFG) and parahippocampal gyrus (PHG) with pathologies such as AD, MCI, Parkinson's and control were selected for analysis. Data from 1,576 participants of the AD Neuroimaging nitiative (ADNI) phases 1, GO and 2 was used for multi-modal neuroimaging analysis. Data from 674 ADNI-1 participants was used for lipidomics analysis. APP/PS1 mice (n = 8, 50% female) and WT mice (n = 8, 50% female) at 7 months old (m.o.) were used for behavioral testing and identifying the effect of fingolimod.
Data exclusions	No data was excluded from the analyses.
Replication	The brain samples used for the study were obtained post-mortem and the results were not replicated for the study. For the mice experiments, 8 biological replications of APP/PS1 and 8 wild-type were used for behavioral testing.
Randomization	Only done for annotation of genetic variants and gene-wide significance thresholds. As SNPs within genes are correlated due to linkage disequilibrium and Bonferroni correction is often too conservative, we used permutation test, which provides a gene-based empirical p-value that corrects for the number of SNPs within each gene by randomly permuting the phenotypes multi times (20,000 times) and performing statistical tests for all permuted data sets.

Blinding	Blinding was not relevant to the study as the pathologies of samples were important for analyses.

Reporting for specific materials, systems and methods

We require information from authors about some types of materials, experimental systems and methods used in many studies. Here, indicate whether each material, system or method listed is relevant to your study. If you are not sure if a list item applies to your research, read the appropriate section before selecting a response.

Materials & experimental systems

n/a	Involved in the study
☒	☐ Antibodies
☒	☐ Eukaryotic cell lines
☒	☐ Palaeontology and archaeology
☐	☒ Animals and other organisms
☒	☐ Clinical data
☒	☐ Dual use research of concern

Methods

n/a	Involved in the study
☒	☐ ChIP-seq
☒	☐ Flow cytometry
☒	☐ MRI-based neuroimaging

Animals and other research organisms

Policy information about studies involving animals; ARRIVE guidelines recommended for reporting animal research, and Sex and Gender in Research

Laboratory animals	APPswe/PS1dE9 (referred to as APP/PS1)
Wild animals	C57Bl/6J mice
Reporting on sex	For all behavioral and synaptic studies at 7 and 9 mo, each experimental group comprised both males and females
Field-collected samples	Plasma samples were collected at two time points (2nd and 4th weeks) after treatment and analyzed by UHPLC and MS-MS.
Ethics oversight	Experiments were approved by the Division of Comparative Medicine (DCM) from SUNY Downstate Medical Center

Note that full information on the approval of the study protocol must also be provided in the manuscript.
